# Correction: The Rsb Phosphoregulatory Network Controls Availability of the Primary Sigma Factor in *Chlamydia trachomatis* and Influences the Kinetics of Growth and Development

**DOI:** 10.1371/journal.ppat.1005309

**Published:** 2015-11-19

**Authors:** The PLOS Pathogens Staff

## Notice of Republication

This article was republished on November 12, 2015, to correct errors that were introduced during the Production process. The line of text included as the legend in Table 1 was incorrectly moved from the Introduction. This text has now been moved back to the Introduction. The word “phosphatase” was also incorrectly added to the “Inter Pro Domain” column in Table 1 and has now been removed. The publisher apologizes for the errors. Please download this article again to view the correct version. The originally published, uncorrected article and the republished, corrected articles are provided here for reference.

## Supporting Information

S1 File Originally published, uncorrected article. (PDF)

S2 File Republished, corrected article. (PDF)
