# Supplementary material for: The Rsb Phosphoregulatory Network Controls Availability of the Primary Sigma Factor in Chlamydia trachomatis and Influences the Kinetics of Growth and Development
Source: PLoS Pathog. 2015 Aug 27;11(8):e1005125. doi: 10.1371/journal.ppat.1005125 (PMC4552016; doi:10.1371/journal.ppat.1005125)
Supplement: S1 Text — (DOCX) [file ppat.1005125.s001.docx]

**Supplementary Methods**

**BACTH Assay**

DHM1 *E. coli* (Δ*cya*), were co-transformed with a pKT25/pKNT25 and pUT18C derived vectors and selected on LB agar supplemented with Carbenicllin (100µg/ml) and Kanamycin (50 µg/ml). Individual clones were randomly selected and used to inoculate overnight cultures of LB broth, supplemented with both antibiotics. The next day, 10µl of broth cultures were used to ‘spot’ LB agar supplemented with antibiotics, X-gal (40µg/ml), and IPTG (0.5mM). Spotted plates were incubated at room temperature (~20˚C) for 3 days. Spots were then dislodged and transferred into 1.25 ml PBS. An aliquot of 0.25 ml was removed to measure the sample OD_600_ and the remaining 1 ml sample was analyzed for b-galactosidase activity via the Miller Assay.

**Protein purification**

Soluble proteins were purified using glutathione sepharose 4B (GE Healthcare) to immobilize GST-tagged proteins. Proteins to be used in their recombinant form were eluted in a buffer of 50 mM Tris-HCl, 10 mM reduced glutathione, pH 8.0. Proteins to be used in their native form were cleaved from the column using Prescision Protease (GE Healthcare) in a buffer of 50 mM Tris HCl, 150 mM NaCl, 1 mM EDTA, 1 mM DTT, pH 7.5. The elution or cleavage buffer was then exchanged to 50 mM Tris-HCl, 150 mM NaCl, 1 mM DTT, pH 7.5 using the Zeba 7kDa MWCO spin desalting column (Thermo Scientific). Samples aliquots were flash-frozen in liquid nitrogen and stored at -80˚C. All proteins were assessed for purity via SDS-PAGE and Coomassie stain. Insoluble proteins (Sigma66) were liberated from the insoluble fraction and immobilized by glutathione sepharose 4B as described in [[1]](https://paperpile.com/c/3xvGO2/toqR). Proteins were eluted or cleaved from the column as described above.

**Surface Plasmon Resonance**

For experiments monitoring the interaction between s-factors and RsbW_Ct_, recombinant purified σ^28^, σ^66^, and σ^54^ were immobilized to flowcells fc2, fc3, and fc4, respectively, of a CM5 sensor chip. GST-RsbV1 S56D (which exhibited minimal binding to RsbW_Ct_) was immobilized in fc1 and used a negative control reference. A series of RsbW_Ct_ concentrations were diluted into running buffer (HEPES 10mM, NaCl 150 mM, and MgCl_2_ 1mM) and sequentially injected through all four flowcells. Response was transformed by the RU observed in the negative control flowcell to yield relative response. For experiments monitoring the interaction of antagonists to RsbW_Ct_, GST-RsbV1, GST-RsbV1 S56A, or GST-RsbV S56D were immobilized to fc2, fc3, and fc4, respectively, on a CM5 sensorchip. Fc1 was left blank and used as reference flowcell. RsbW_Ct_ was injected at a concentration of 2.5µM in running buffer, in the absence or presence of 0.5mM ATP (added immediately prior to injection). Relative response was calculated as the difference between sample flowcells and the reference (no ligand) flow cell. Procedure was repeated on a separate CM5 sensorchip for RsbV2 and its derivatives.

**Kinase and phosphatase assays**

For kinase assays, approximately 2.5µM of GST-RsbV1 or GST-RsbV2 were incubated in a reaction buffer of 25mM Tris HCl, 25mM KCl, 0.5mM DTT and 1.5mM MgCl_2_, pH 7.5. Prior to addition of RsbW_Ct_, an aliquot was removed and combined with an equal volume of 2X Laemmli buffer (8% glycerol, 4% SDS, 120mM Tris pH 6.8, 0.4% Bromophenol Blue, 10% b-mercaptoethanol). The remaining reaction was pre-warmed to 30˚C, RsbW_Ct_ added at to a final concentration of 250nM (1:10 enzyme to substrate), and the reaction initiated by 1mM ATP. Aliquots were removed, denatured in Laemmli buffer at 10, 30, 60 and 120 minutes, and resolved on a 12% tris-glycine-SDS (TGS) polyacrylamide gel supplemented with 20µM Phos-tag reagent (Alpha Laboratories) and 100µM MnCl_2._ Protein migration was visualized by Silver stain (ProteoSilver Silver Stain Kit; Sigma). For phosphatase assays, recombinant, purified GST-RsbV1 or GST-RsbV2 were phosphorylated overnight in an excess of RsbW_Ct_ and ATP, bound to a glutathione Sepharose 4B resin, separated from the non-tagged RsbW_Ct_ and cleaved off the resin as described in the protein purification section. Phospho-RsbV1 or phospho-RsbV2 were then diluted to a concentration of 1.25µM in a reaction buffer composed of 50mM Tris-HCl, 50mM KCl, 10mM MgCl_2_, 1mM MnCl_2_, pH 7.5. The reaction was pre-warmed to 30˚C and a 10µl sample was removed (t=0) prior to initiation of the reaction via the addition of C-RsbU_Ct_ or CT259 at a final concentration of 250 nM (5:1 substrate to enzyme). Aliquots were removed at 10 and 60 minutes, denatured in an equivalent volume of 2X Laemmli buffer, and resolved on a Phos-tag supplemented 15% TGS-polyacrylamide gel as described.

**Transformation**

1.5 µl of 1M CaCl_2_ was added to 1 µg of plasmid and the volume filled to 29µl with 25mM Tris-HCl, pH=7.5. 1µl of *C. trachomatis* serovar L2/25667R (~5x10^9^ IFU/ml) was added, and the reaction was mixed by pipetting up and down 10 times. The transformation was incubated at 25˚C on a heat-block for 25 minutes. The entire reaction was then diluted into 2ml HBSS, and then used to inoculate 1well of a 6well cluster plate containing a confluent HeLa cell monolayer. The plate was centrifuged for 1 hour at 20˚C at 500*g. The inoculum was aspirated and the infected monolayer incubated in DMEM supplemented with 10% fetal bovine serum (FBS), 1µg/ml cycloheximide for 32 hours at 37˚C, 5% CO_2_. Infected monolayers were then dislodged into 1ml 10% PBS, vortexed extensively with sterile glass beads, and then diluted into 14ml PBS (100%). This inocula was used to infect 3x15cm tissue culture dishes containing 70-90% confluent HeLa monolayers. Infected cultures were incubated for 2 days at 37˚C, 5% CO_2_ in 25ml of DMEM supplemented with 10% FBS, 1µg/ml cycloheximide, and 1U/ml penicillin G.. The infected monolayer of each dish was then dislodged into 3ml 10% PBS. The cell suspensions were pooled and vortexed extensively with sterile glass beads. The 9ml total suspension was added to 9ml of HBSS, and the total volume was used to inoculate 1x 15cm tissue culture dish of confluent HeLa cells. After 2 hours of invasion at 37˚C, inoculum was aspirated and the infected monolayer incubated in a selective medium as before. At confluency, infected monolayers were trypsinized and 50% passaged into a new 15cm tissue culture dish in DMEM supplemented with 10%FBS and 1U/ml penicillin. Cultures were supplemented with cycloheximide the next day after cells had attached. This process was continued until transformants were evident.

Transformants were then plaque purified, propagated and harvested. Penicillin Inhibitory Concentration (IC) curves were generated by incubation of infected HeLa monolayers in the indicated Penicillin concentration for 42 hours (treated immediately post-infection), prior to sample harvest and IFU quantification as described below. The mock-supplemented control (no Penicillin) was used to normalize data represented in **Figure S4B**.

1. [Tao H, Liu W, Simmons BN, Harris HK, Cox TC, Massiah MA. Purifying natively folded proteins from inclusion bodies using sarkosyl, Triton X-100, and CHAPS. Biotechniques. 2010;48: 61–64.](http://paperpile.com/b/3xvGO2/toqR)
