# Supplementary figures and images for: The Rsb Phosphoregulatory Network Controls Availability of the Primary Sigma Factor in Chlamydia trachomatis and Influences the Kinetics of Growth and Development
Source: PLoS Pathog. 2015 Aug 27;11(8):e1005125. doi: 10.1371/journal.ppat.1005125 (PMC4552016; doi:10.1371/journal.ppat.1005125)

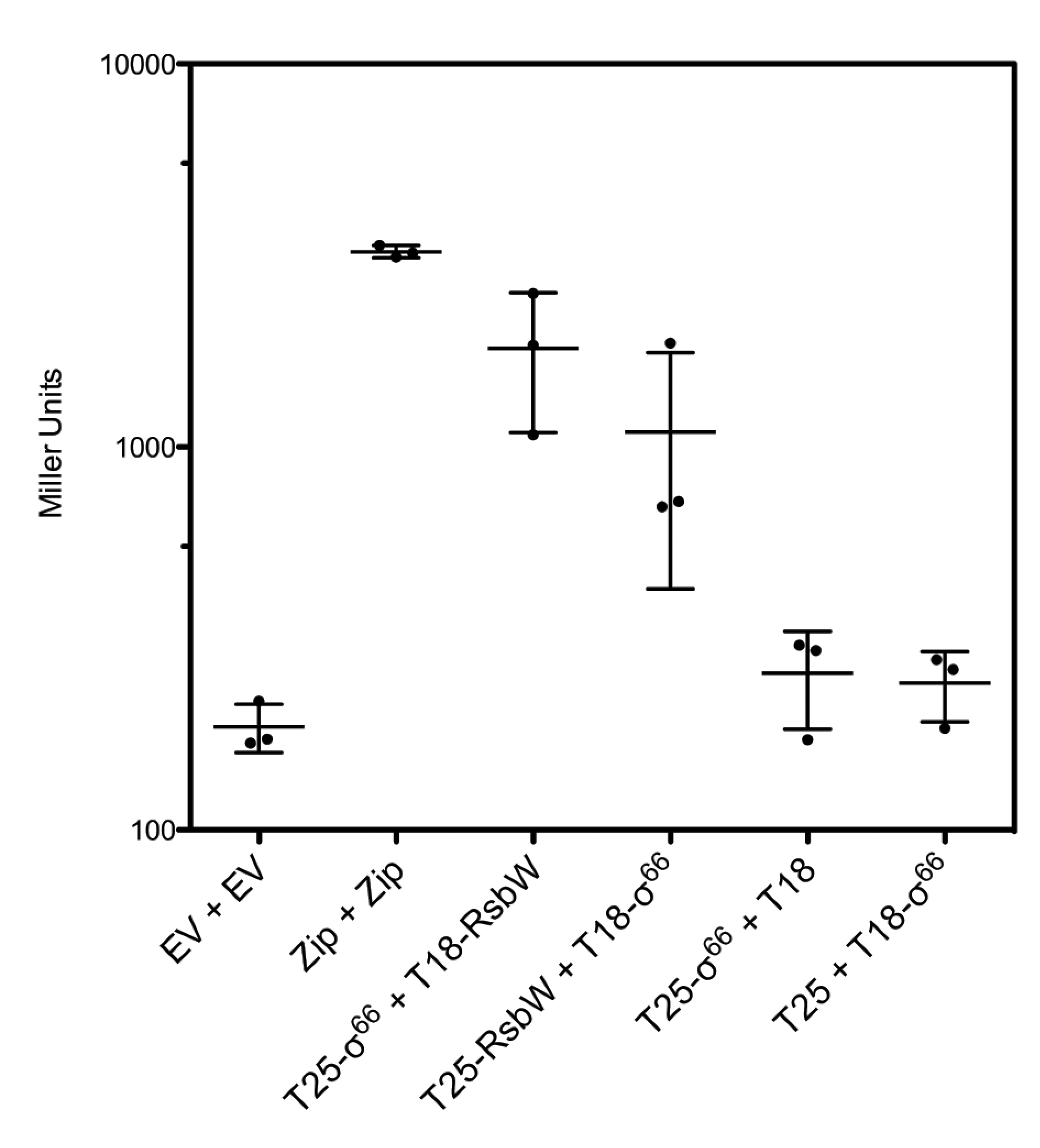

Supplement: S1 Fig — The BACTH assay was performed with additional controls to ensure that activation of the cAMP dependent promoter was due to reconstitution of the AC enzyme, and not artificial activation via the heterologous expression of σ66 in DHM1 E. coli. Each data point represents the measured LacZ activity of an expanded co-transformant that had been spotted for 2 days. (TIF) [file ppat.1005125.s006.tif]

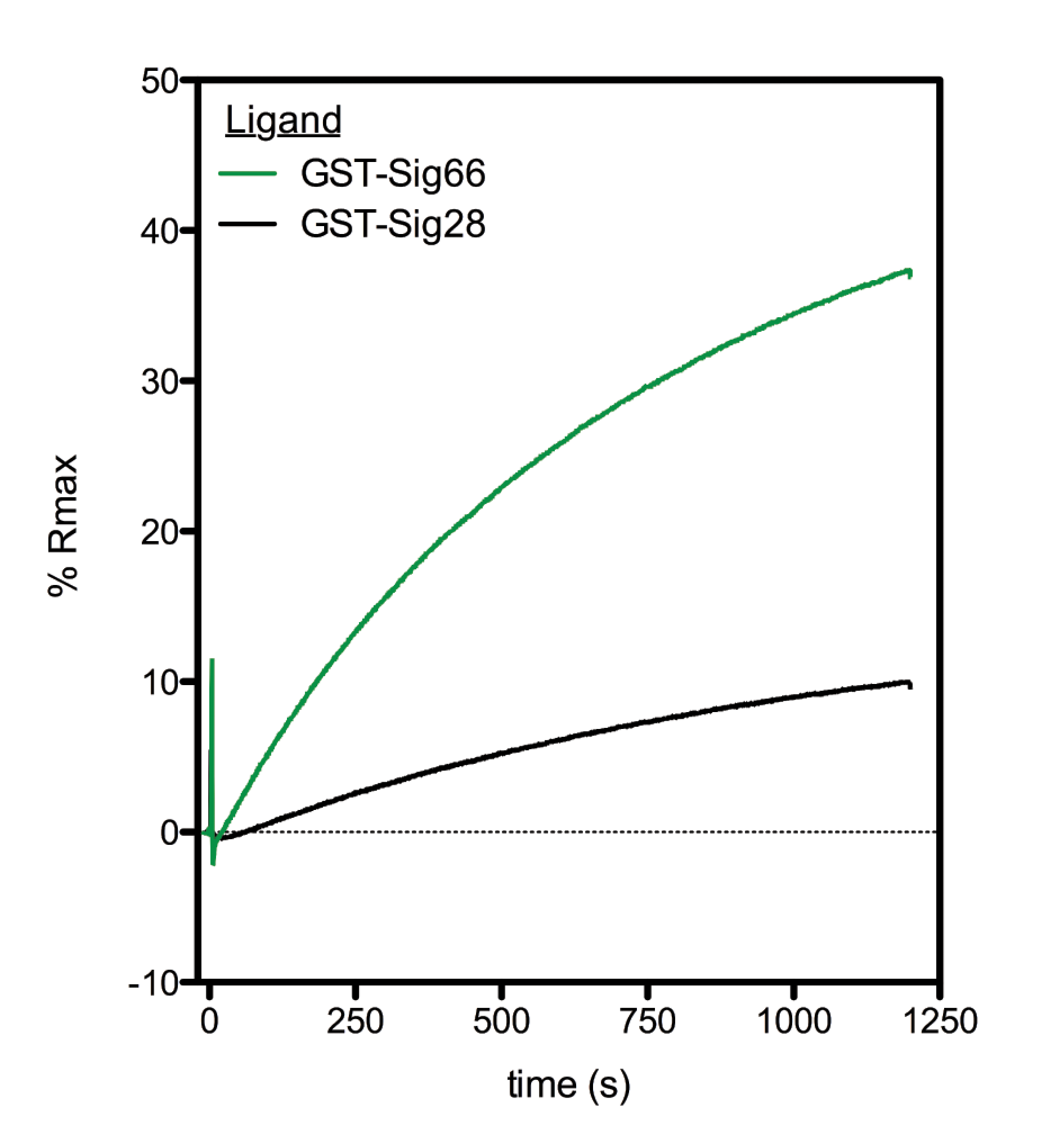

Supplement: S2 Fig — SPR analysis of captured ligands. Immunoglobulin targeting glutathione-s-transferase (GST) was immobilized to all flow cells of a CM5 sensorchip. GST-σ66 and GST-σ28 were captured in each flow cell, prior to a GST blocking step (which created a GST only reference flowcell), and analyte charge of RsbWCt at a concentration of 5000nM. The GST-only flow cell was used as a reference, and the relative response was transformed by the maximum binding theoretically possible (Rmax) based on the amount of ligand capture. (TIF) [file ppat.1005125.s007.tif]

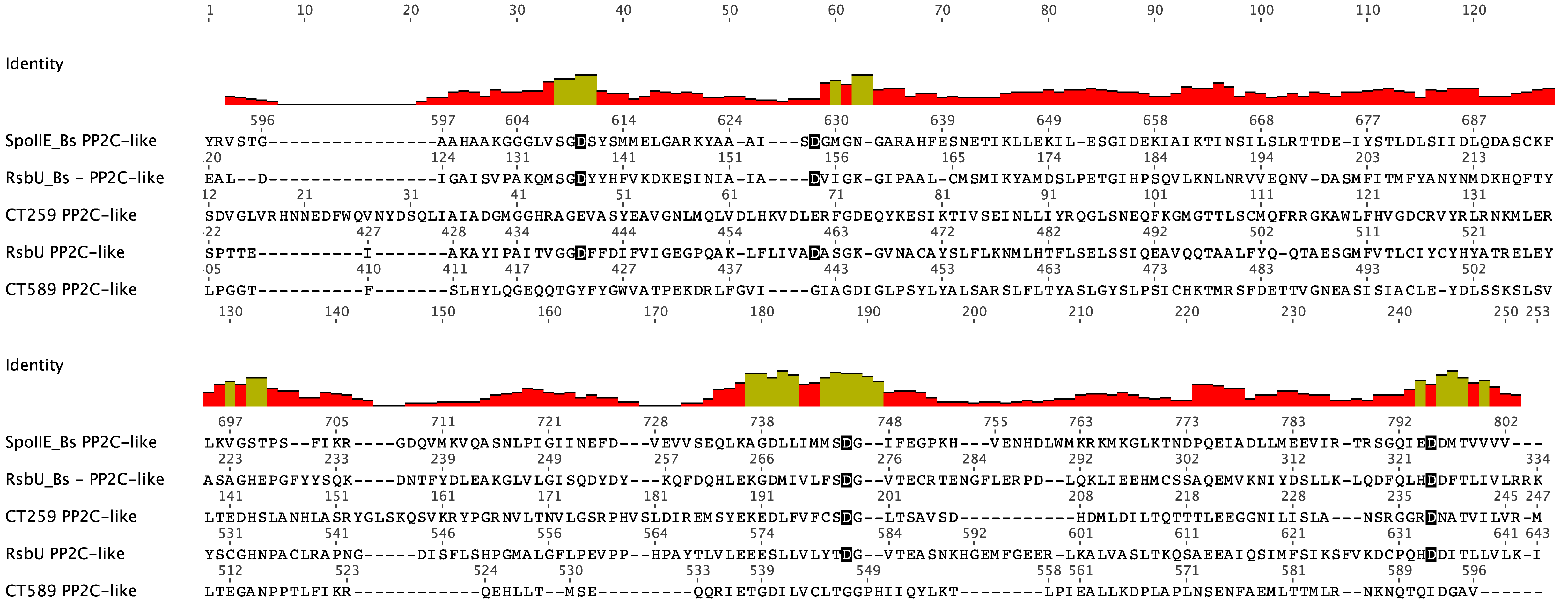

Supplement: S3 Fig — The PP2C-like domains for the three chlamydial proteins were extracted and aligned with the PP2C-like domains of SpoIIE and RsbU from B. subtilis (RsbU_Bs). CT589 does not conserve residues necessary for Mn2+/Mg2+ coordination, which are essential for phosphatase activity in PP2C-like phosphatases. CT259 is conserved at positions D199 and D238 (corresponding to D582 and D634 in RsbUCt), and exhibits conservative mutations at positions E27 and E69 (corresponding to D440 and D461 in RsbUCt), allowing theoretical ability to coordinate the Mn2+/Mg2+ ions that are required for phosphatase activity. (TIF) [file ppat.1005125.s008.tif]

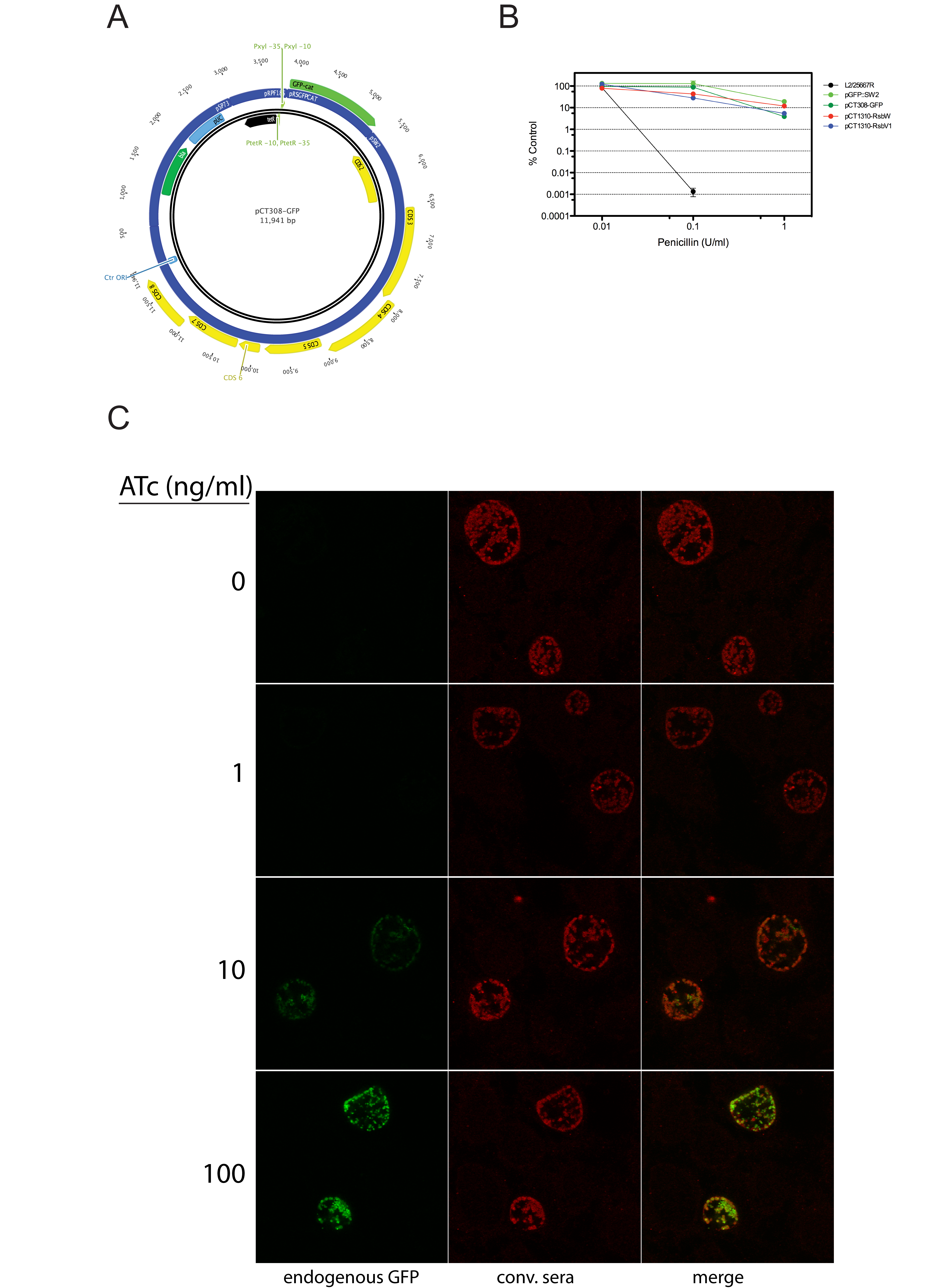

Supplement: S4 Fig — Characterization of expression shuttle vectors. A) Plasmid pCT308-GFP was generated by replacing the promoter driving the gfp-cat cassette from pGFP::SW2 with the tetracycline inducible promoter system from pRPF185. pCT1310-RsbW and pCT1310-RsbV1 were made by exchanging the gfp-cat cassette with the genetic sequence of corresponding genes out of C. trachomatis gDNA Serovar D/UW/Cx. B) Penicillin inhibitory concentration curves were generated for stable, plaque purified transformant strains. Infections were incubated in media containing the indicated final concentration of Penicillin for 42 hours, prior to cell disruption and quantification of recoverable infectious progeny. All three transformant strains were as resistant to penicillin as the positive control pGFP::SW2. C) The pCT308-GFP strain was examined for fluorescence in non-supplemented and supplemented media (0, 1, 10, or 100 ng/ml ATc). Samples were (mock-) induced at 6 hours post infection and fixed at 28 hours post infection. Chlamydia was immunolabeled using convalescent human sera and a DyLight 594-conjugated secondary antibody. Images were captured by confocal microscope as described in the methods section. (TIF) [file ppat.1005125.s009.tif]

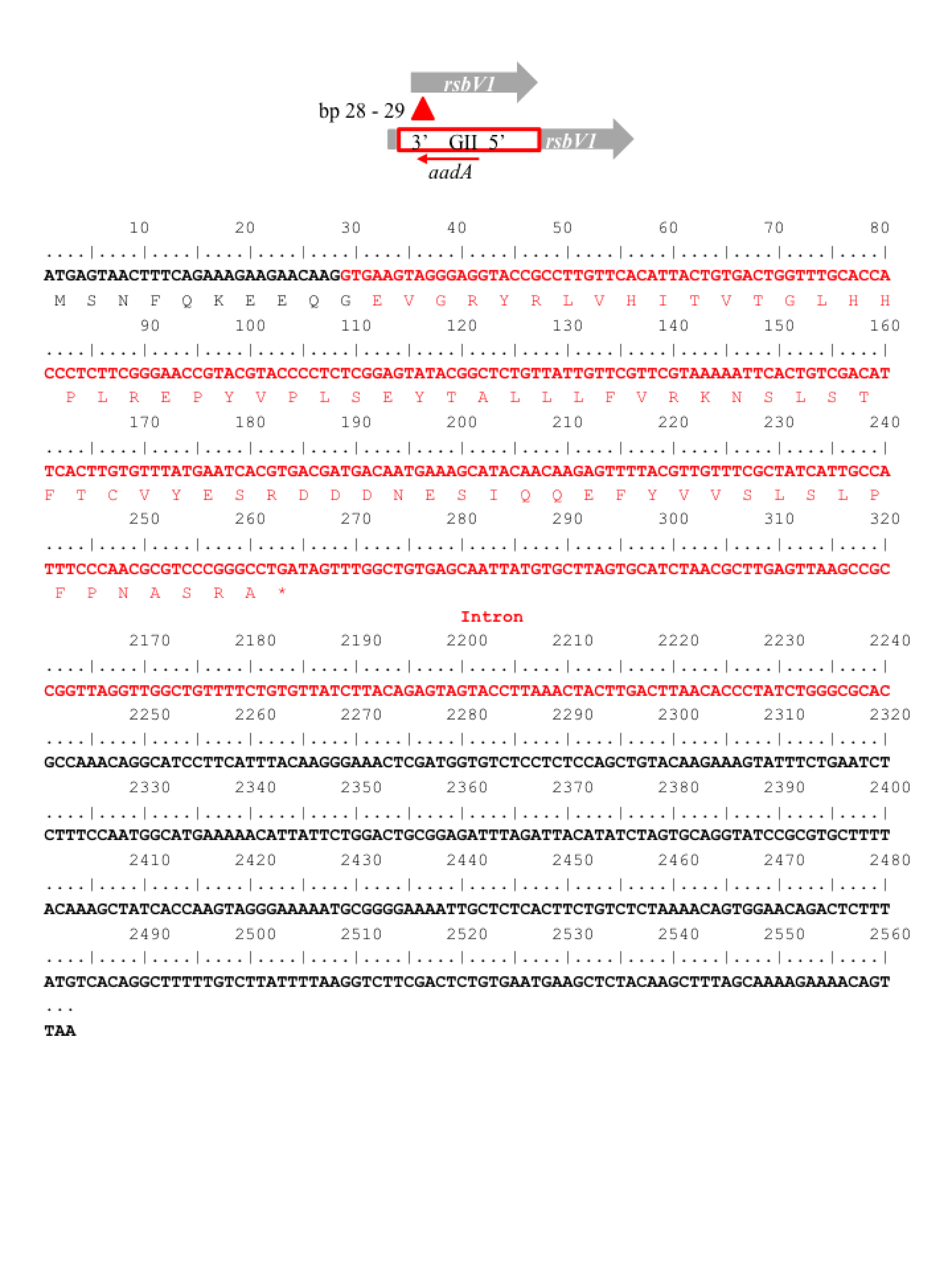

Supplement: S5 Fig — The rsbV1 and rsbv1::GII(aadA) loci were amplified via PCR and cloned into pJET for Sanger sequencing. The intron inserted in an anti-sense orientation (relative to rsbV1) at position 28 (A in ATG designated as position 1) resulting in alteration of the wild type ORF after ten amino acids and a stop codon after 87 amino acids. The wild type RsbV1 is 116 amino acids of which only the first ten would be present in the “recombinant” protein, if produced. The GII intron sequence is shown in red and is truncated for brevity. (TIF) [file ppat.1005125.s010.tif]

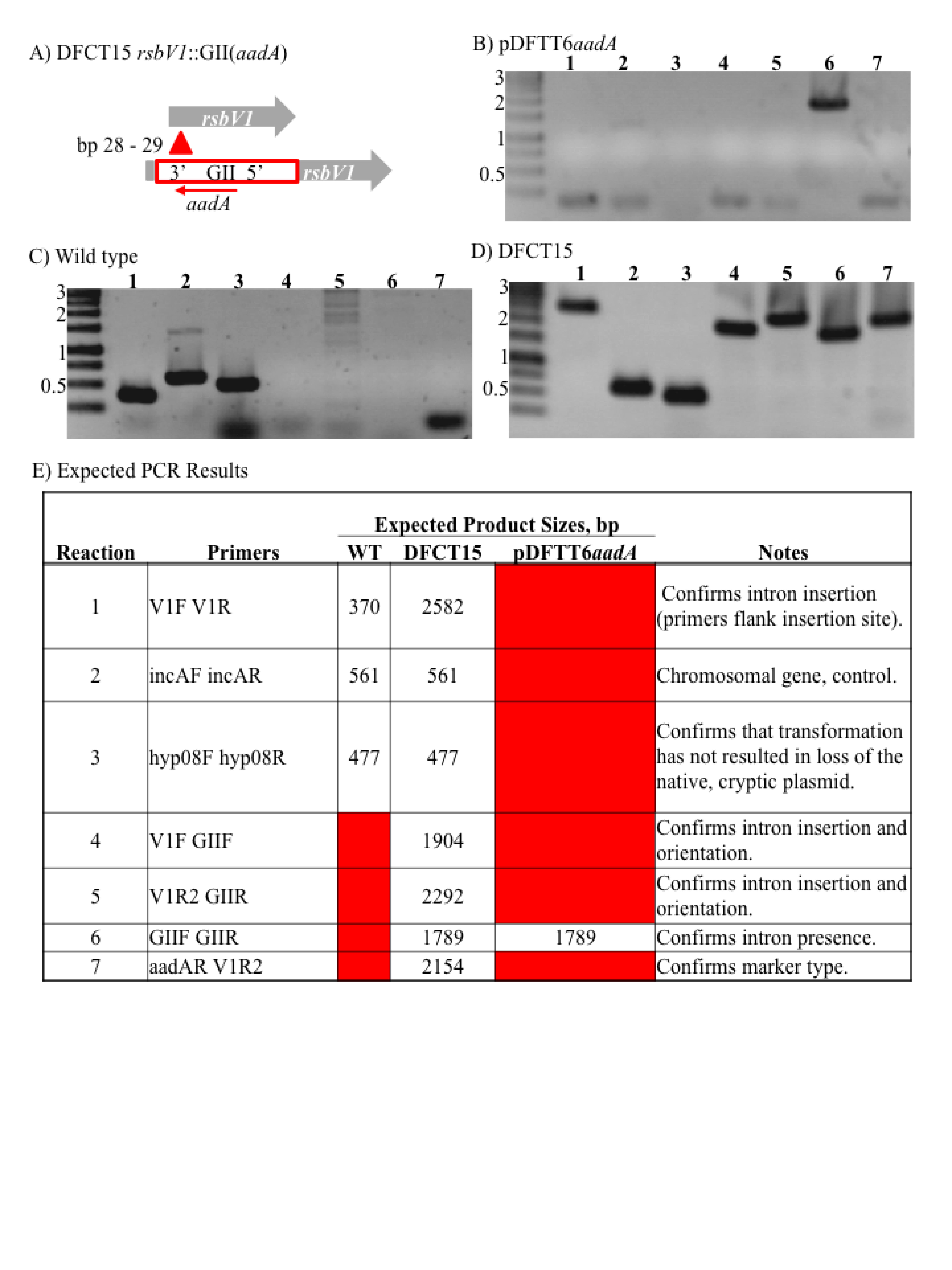

Supplement: S6 Fig — PCR was used to confirm intron insertion and orientation. Loci maps are shown in A and PCR results are shown for the intron-donor vector (B), wild type strain (C), and DFCT15 (D). Expected products for each reaction are shown in (E). PCR reactions were performed with 50 ng of genomic DNA (wild type strain and DFCT15) or 1 ng of purified plasmid DNA (pDFTT6aadA). PCR products were run on 0.8% agarose gels, stained with ethidium bromide, and visualized using UV trans-illumination. Images were inverted to improve contrast. Molecular weight markers (in kbp) are shown to the left of each gel image. (TIF) [file ppat.1005125.s011.tif]

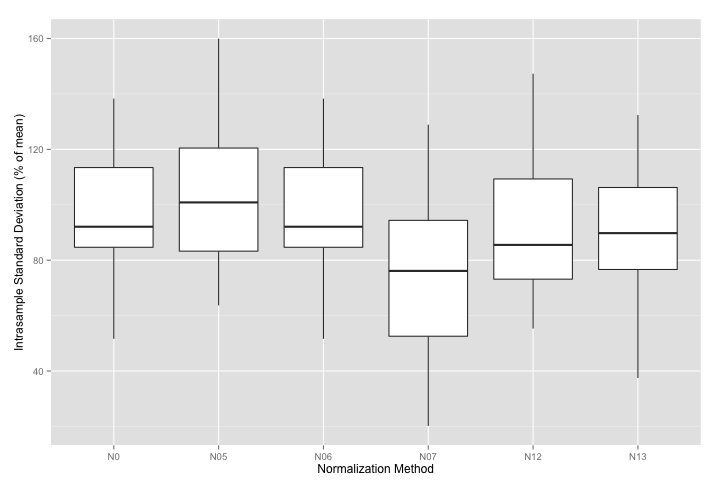

Supplement: S7 Fig — We analyzed the ‘intrasample’ standard deviation from biological replicates (i.e. same strain, time-point harvest, and target gene- ompA, 16S rRNA, and hctB only) upon various normalization strategies. We reasoned that the best normalization strategy would result in the lowest mean of intrasample standard deviation. Standard deviations were converted to % of mean for ease of comparison. Boxplots of the intrasample standard deviation are shown for the primary data (N0) and after various strategies (N05, gDNA of parallel within the subset; N06, mean gDNA of all parallel subsets; N07, gapdh; N12, geometric mean N07 and N05; N13, geometric mean of N07 and N06). The lowest cumulative intra sample standard deviation occurred when expression data was normalized by the host housekeeping reference gene, gapdh (N07). However this strategy failed to account for the number of chlamydiae present within each sample. Therefore we chose to normalize by strategy N12, which is the geometric combination of host gapdh expression and the C. trachomatis gDNA measured from a parallel sample. (TIFF) [file ppat.1005125.s012.tiff]

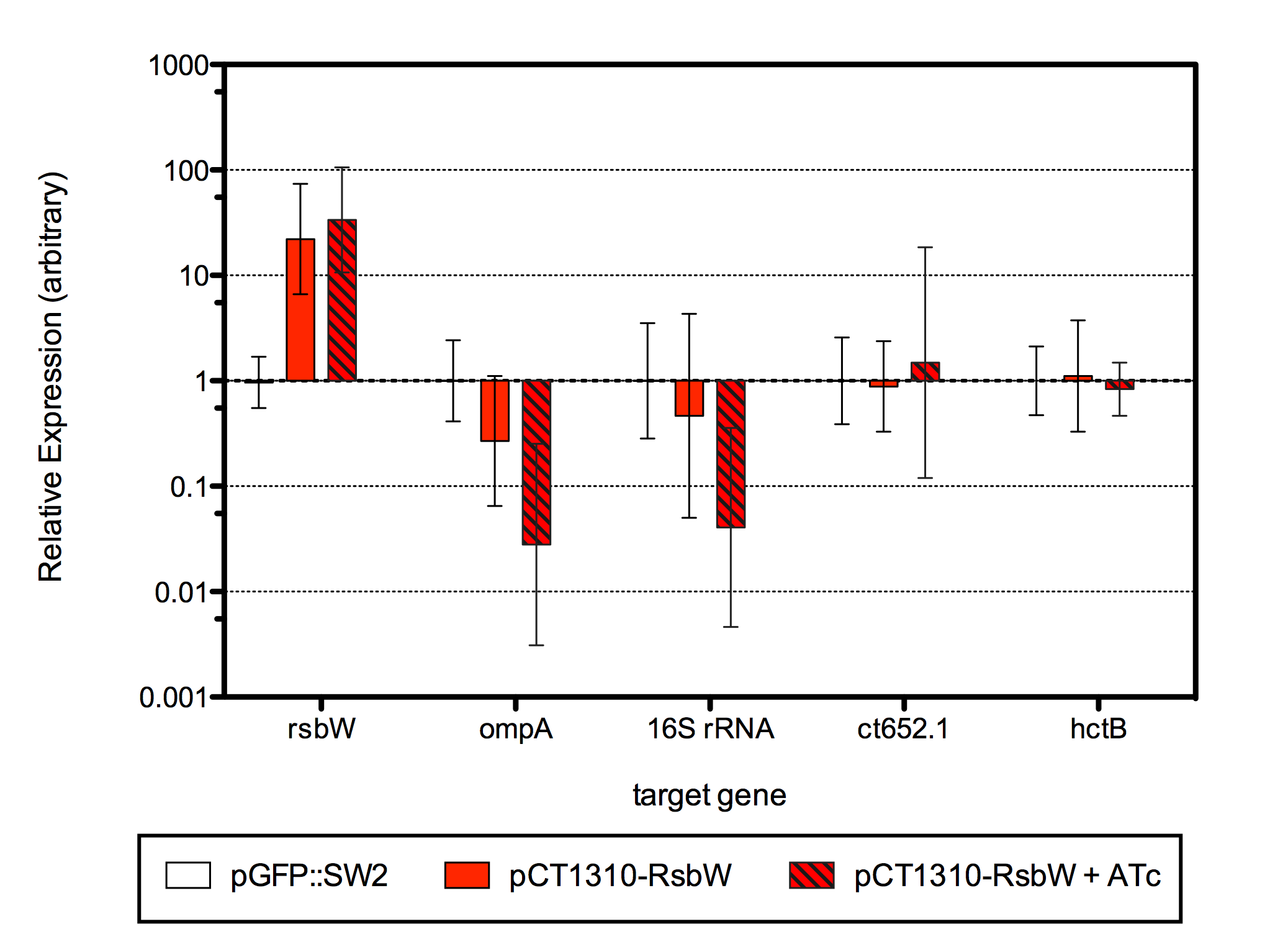

Supplement: S8 Fig — Transcript levels of two σ66-dependent genes (16S rRNA and ompA), one putative σ54-dependent gene (ct652.1), and one σ28-dependent gene (hctB) from strain L2/25667R pCT1310-RsbW were monitored via qPCR, normalized to exogenous gDNA controls, and then calibrated to the normalized levels of a control strain (L2/25667R pGFP::SW2). Bars represent the geometric mean of relative expression from time-points collected during 14 to 18 hours post infection (prior to typical EB redifferentiation). Error bars represent the 95% confidence intervals. Elevated rsbW expression was concomitant with decreased expression of σ66-transcribed genes, whereas genes transcribed by the alternative σ-factors were not differentially regulated. (TIFF) [file ppat.1005125.s013.tiff]

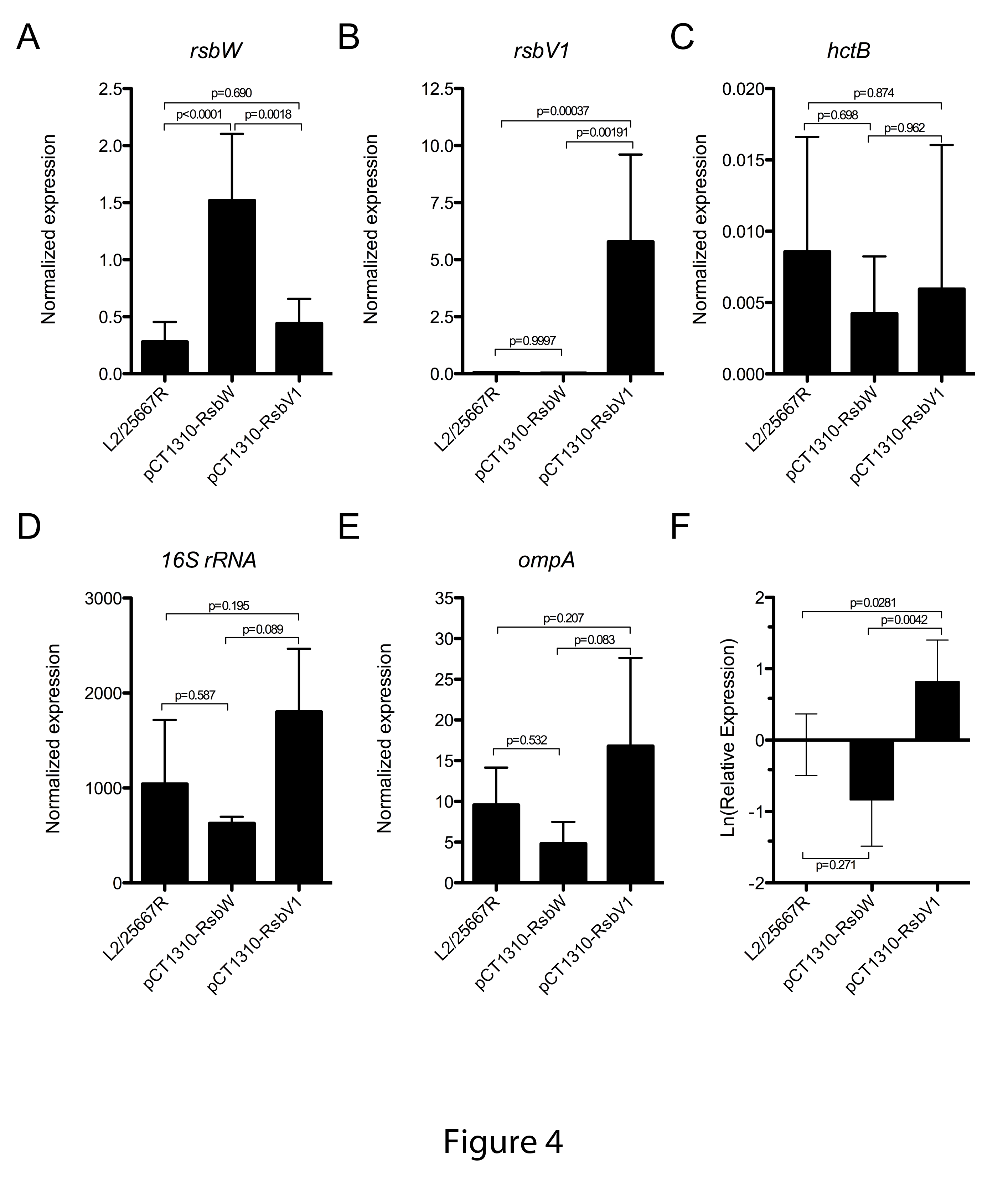

Supplement: S9 Fig — Transcript levels (rsbW, A; rsbV1, B; hctB, C; 16S rRNA, D; ompA, E) were measured at 18 hours post infection in C. trachomatis L2/25667R (plasmid-free) and daughter strains harboring shuttle vectors, pCT1310-RsbW or pCT1310-RsbV1. Graphs show the expression normalized by an exogenous genomic DNA control. Relative levels of σ66-dependent gene transcripts, 16S rRNA and ompA, were reduced upon rsbW expression and increased upon rsbV1 expression (combined mean relative expression shown in F), whereas a σ28-dependent transcript, hctB, was not altered by ectopic expression of either cassette. Error bars represent the 95% confidence interval. Statistical p-values are derived from One-way ANOVA with Tukey’s multiple comparisons post-test performed in R. (TIF) [file ppat.1005125.s014.tif]

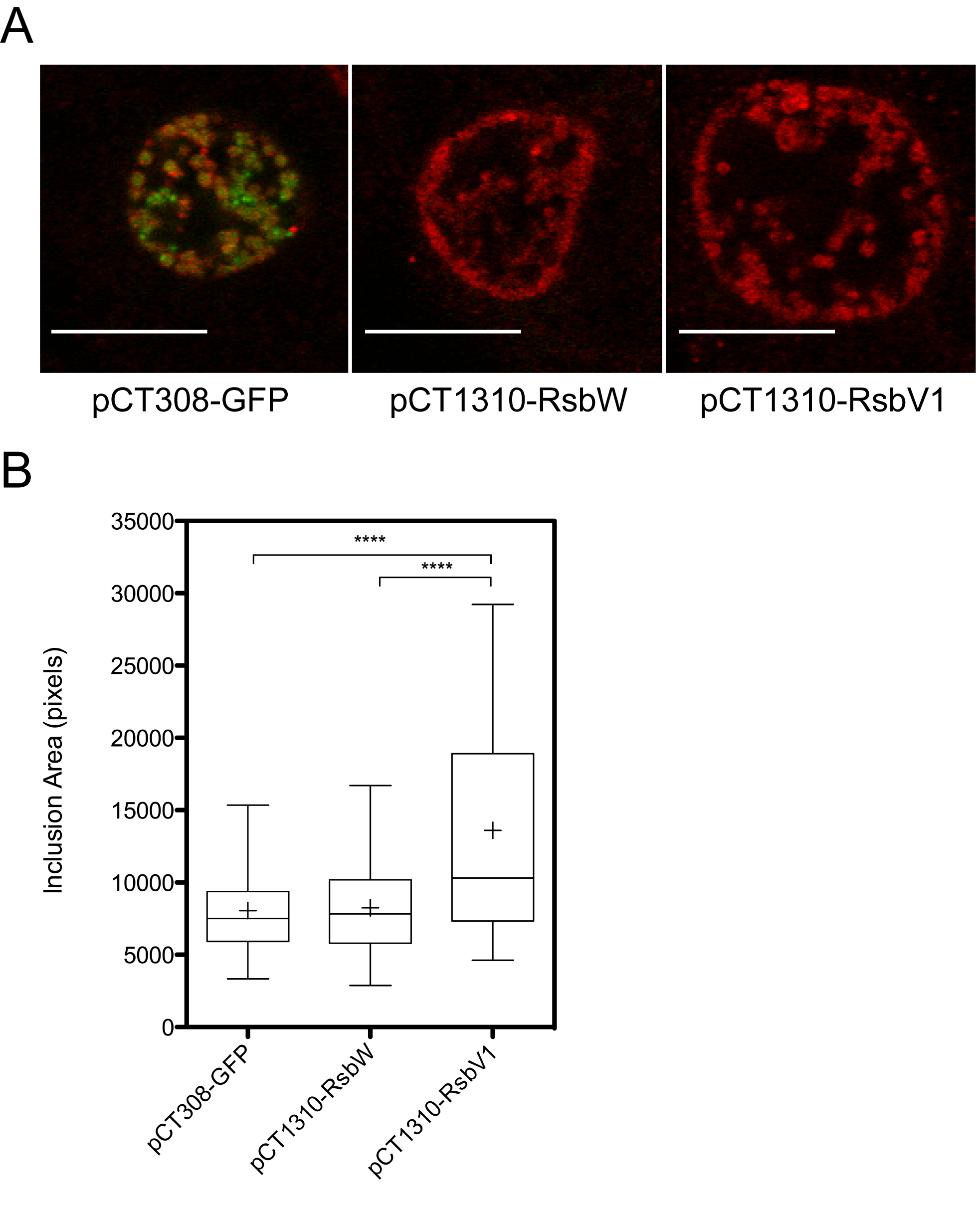

Supplement: S10 Fig — Chlamydia trachomatis morphology was examined in L2/25667R transformed strains harboring pCT308-GFP, pCT1310-RsbW, or pCT1310-RsbV1 (A). Samples were fixed at 28 hours post infection and images were captured using Leica SP5 confocal microscope in both red (DyLight-594 immunolabeled Chlamydia) and green (endogenous GFP expression) channels. Inclusions representative of the mean inclusion area are shown. Scale bars represent 10 μm. Box and Whisker plots representing inclusion area from confocal micrographs are shown in (B). Each box represents the upper/lower quartiles transected by the median inclusion size. Whiskers represent the 5–95% confidence interval, and (+) represent the mean of each group. Statistical p values are derived from One-way ANOVA with Tukey’s multiple comparison post-test (**** represents p<0.0001). (TIF) [file ppat.1005125.s015.tif]
