# Supplementary material for: Correction: The Rsb Phosphoregulatory Network Controls Availability of the Primary Sigma Factor in Chlamydia trachomatis and Influences the Kinetics of Growth and Development
Source: PLoS Pathog. 2015 Nov 19;11(11):e1005309. doi: 10.1371/journal.ppat.1005309 (PMC4653039; doi:10.1371/journal.ppat.1005309)
Supplement: S1 File — (PDF) [file ppat.1005309.s001.pdf]

PLOS | PATHOGENS

RESEARCH ARTICLE

The Rsb Phosphoregulatory Network Controls Availability of the Primary Sigma Factor in *Chlamydia trachomatis* and Influences the Kinetics of Growth and Development

Christopher C. Thompson[1], Cherry Griffiths[1], Sophie S. Nicod[2], Nicole M. Lowden[3], Sivaramesh Wigneshweraraj[2], Derek J. Fisher[3], Myra O. McClure[1]*

1 Jefferiss Trust Laboratories, Faculty of Medicine, Imperial College London, St. Mary's Hospital Campus, London, United Kingdom, **2** MRC Centre for Molecular Bacteriology and Infection, Imperial College London, London, United Kingdom, **3** Department of Microbiology, Southern Illinois University, Carbondale, Carbondale, Illinois, United States of America

* m.mcclure@imperial.ac.uk

CrossMark
click for updates

OPEN ACCESS

Citation: Thompson CC, Griffiths C, Nicod SS, Lowden NM, Wigneshweraraj S, Fisher DJ, et al. (2015) The Rsb Phosphoregulatory Network Controls Availability of the Primary Sigma Factor in *Chlamydia trachomatis* and Influences the Kinetics of Growth and Development. PLoS Pathog 11(8): e1005125. doi:10.1371/journal.ppat.1005125

Editor: Isabelle Derré, University of Virginia School of Medicine, UNITED STATES

Received: March 4, 2015

Accepted: August 3, 2015

Published: August 27, 2015

Copyright: © 2015 Thompson et al. This is an open access article distributed under the terms of the Creative Commons Attribution License, which permits unrestricted use, distribution, and reproduction in any medium, provided the original author and source are credited.

Data Availability Statement: The primary data for Fig 1 is available from the FigShare database (10.6084/m9.figshare.1320908). The primary data for Fig 2 is available from the FigShare database (10.6084/m9.figshare.1320906). The primary data for 1-step growth, 2-step growth and gene expression summarized in Figs 4 and 5 are available in the S1 Dataset in the supplementary information. The R analysis script for these portions of the figures is available from the FigShare database (10.6084/m9.figshare.1466906). The primary data for plaque

Abstract

Chlamydia trachomatis is an obligate intracellular human pathogen that exhibits stage-specific gene transcription throughout a biphasic developmental cycle. The mechanisms that control modulation in transcription and associated phenotypic changes are poorly understood. This study provides evidence that a switch-protein kinase regulatory network controls availability of σ^{66}, the main sigma subunit for transcription in *Chlamydia*. *In vitro* analysis revealed that a putative switch-protein kinase regulator, RsbW, is capable of interacting directly with σ^{66}, as well as phosphorylating its own antagonist, RsbV1, rendering it inactive. Conversely, the putative PP2C-like phosphatase domain of chlamydial RsbU was capable of reverting RsbV1 into its active state. Recent advances in genetic manipulation of *Chlamydia* were employed to inactivate *rsbV1*, as well as to increase the expression levels of *rsbW* or *rsbV1*, *in vivo*. Representative σ^{66}-dependent gene transcription was repressed in the absence of *rsbV1* or upon increased expression of RsbW, and increased upon elevated expression of RsbV1. These effects on housekeeping transcription were also correlated to several measures of growth and development. A model is proposed where the relative levels of active antagonist (RsbV1) and switch-protein anti-sigma factor (RsbW) control the availability of σ^{66} and subsequently act as a molecular 'throttle' for *Chlamydia* growth and development.

Author Summary

Chlamydia trachomatis is the leading cause of both bacterial sexually transmitted infection and infection-derived blindness world-wide. No vaccine has proven protective to date in humans. *C. trachomatis* only replicates from inside a host cell, and has evolved to acquire a variety of nutrients directly from its host. However, a typical human immune response

expansion measurement is available from the FigShare database (10.6084/m9.figshare.1473082). The R analysis script for plaque expansion measurement is available from the FigShare database (10.6084/m9.figshare.1466907). The primary imaging files for transformant strain analysis (S10 Fig) is available from the FigShare database (10.6084/m9.figshare.1309627).

Funding: This report was funded by a post-doctoral research fellowship award from the Jefferiss Research Trust (London, UK) and by National Institutes of Health, 1R15AI109566-01A1 and 1R21AI115238-01. The funders had no role in study design, data collection and analysis, decision to publish, or preparation of the manuscript.

Competing Interests: The authors have declared that no competing interests exist.

will normally limit the availability of a variety of essential nutrients. Thus, it is thought that the success of *C. trachomatis* as a human pathogen may lie in its ability to survive these immunological stress situations by slowing growth and development until conditions in the cell have improved. This mode of growth is known as *persistence* and how *C. trachomatis* senses stress and responds in this manner is an important area of research. Our report characterizes a complete signaling module, the Rsb network, that is capable of controlling the growth rate or infectivity of *Chlamydia*. By manipulating the levels of different pathway components, we were able to accelerate and restrict the growth and development of this pathogen. Our results suggest a mechanism by which *Chlamydia* can tailor its growth rate to the conditions within the host cell. The disruption of this pathway could generate a strain incapable of surviving a typical human immune response and would represent an attractive candidate as an attenuated growth vaccine.

Introduction

Chlamydia trachomatis is the leading cause of bacterial sexually transmitted infection worldwide [1], as well as the leading cause of infection-associated blindness [2]. Members of the *Chlamydiaceae* are obligate intracellular parasites that must complete a unique intracellular development cycle in order to propagate. This cycle is characterized by phenotypic variation between an Elementary Body (EB) that is infectious but metabolically quiescent, and a Reticulate Body (RB) that is replicative but not infectious. Cellular infection is initiated by the EB, which attaches and induces its own intake via translocation of effector cargo. Once inside the cell, the EB differentiates into an RB, which replicates via binary fission. During the late stages of infection, RBs asynchronously re-differentiate into the EB form (reviewed in [3]). Alternatively, certain stress conditions mediate the onset of a separate growth mode (termed persistence), which was defined as a 'viable but non-cultivable' state, in which chlamydiae fail to complete the development cycle and instead differentiate into aberrant, enlarged particles; this phenotype is reversible upon abatement of the mediating stress [4]. While modulations in transcript levels are associated with the stages of acute development [5–7] and the onset of persistence [8,9], the regulatory mechanisms that govern these phenotypic shifts are not understood.

Sigma factors are responsible for the recruitment of the core RNA polymerase (RNAP) to cognate promoter elements, and, thus, their function dictates the subset of genes transcribed within a cell. *Chlamydia* encodes three sigma factors, σ^{66}, σ^{54}, and σ^{28}, whose individual expression patterns [10–12] fail explain the stage-specific transcription profiles observed in acute chlamydial development [12]. Assuming these sigma factors participate in distinct functions, then post-expression mechanisms of regulation must theoretically be employed.

Switch-protein kinase modules are common effectors of energy and stress responses in bacteria. One of the best studied is the 'Regulator of SigmaB' or Rsb system in *Bacillus subtilis*. Within this type of module, a component called the switch-protein can either bind to and affect the function of a target protein, or function as a kinase in the phosphorylation of a network antagonist. Phosphorylation of the antagonist prevents further interaction with the switch-protein. The function of a competing PP2C-like phosphatase controls the level of active antagonist. In the absence of active (*i.e.* non phosphorylated) antagonist, the switch protein is driven towards its regulatory function. Commonly, the target of the switch-protein is a sigma factor, as is the case for the RsbW protein in *B. subtilis* (reviewed in [13]). Analogues of a switch-protein kinase regulatory module are conserved in the *C. trachomatis* genome [14] and were named after the *B. subtilis* module.

Table 1. Rsb analogues in the *Chlamydiaceae*.

Inter Pro Domain	Function	Name	Notes:
IPR003658	Anti-anti-sigma factor	RsbV1	pI = 5.05; Ser56 accepts phosphate [17]
		RsbV2	pI = 8.04; Ser55 accepts phosphate [17]
IPR003594	Histidine kinase-like ATP-binding domain	$RsbW_{Ct}$	phosphorylates RsbV1 and RsbV2 [17]; switch regulatory function unknown at study onset
IPR001932	PP2C-like phosphatase	$RsbU_{Ct}$	transverses membrane; HAMP linker
phosphatase		CT589	transverses membrane; lacks essential residues for metal coordination and phosphatase activity
		CT259	cytosolic; metal coordinating residues conserved

The current study expands on these works, and further outlines the functions of Rsb module members in *Chlamydia*. *In vitro* and *in vivo* evidence indicate that the regulatory target of $RsbW_{Ct}$ is the primary σ-factor for *Chlamydia*, σ^{66}, and that opposing functions of $RsbW_{Ct}$ and the antagonist, RsbV1, contribute to the amount of σ^{66} that is available for association with the core RNAP complex.

doi:10.1371/journal.ppat.1005125.t001

Putative components of the Rsb switch-protein regulatory system, outlined in **Table 1**, include one analogue of the switch-protein kinase (RsbW), two analogues of the module antagonist (RsbV1 and RsbV2), and three proteins that contain recognized PP2C-like phosphatase domains (RsbU, CT589, and CT259) [14]. Henceforth, chlamydial analogues with common family-member protein names will be designated with a 'Ct' subscript, *e.g.* $RsbW_{Ct}$. Previous studies have revealed that all potential module members are expressed [12,15] and that $RsbW_{Ct}$ is a kinase specific for RsbV1 and RsbV2- although a 'switch' function regulatory target has not been identified [16,17].

Results

RsbW associates directly with σ^{66}

The regulated target of the putative switch-protein kinase analogue in *Chlamydia* ($RsbW_{Ct}$) had not been identified at the onset of this study. Therefore, a bacterial adenylate cyclase two-hybrid (BACTH) system [18] was employed to screen for interactions between $RsbW_{Ct}$ and the three chlamydial sigma factors: σ^{28}, σ^{54}, and σ^{66} (**Fig 1A**). In order to gauge the relevance of this method for the screening of RsbW-type interactions, homologous proteins of known function from *B. subtilis* [19,20] were used as positive (σ^B + $RsbW_{Bs}$) and negative (σ^B + $RsbT_{Bs}$) control combinations. As expected, interaction of σ^B with its cognate anti-sigma factor, $RsbW_{-Bs}$, complemented adenylate cyclase (AC) activity, and expression of the cAMP-dependent *lacZ* reporter cassette was detected via the Miller Assay. In contrast, co-expression of σ^B with $RsbT_{Bs}$ (a paralogue of $RsbW_{Bs}$ that does not bind to σ^B) did not exhibit complementation. When $RsbW_{Ct}$ was expressed with the chlamydial alternative sigma factors, σ^{28} and σ^{54}, similar activities to the empty vector controls were observed (t = 0.124 and t = 0.023, respectively; One-way ANOVA, Bonferroni's multiple comparison post test). In contrast, an interaction between $RsbW_{Ct}$ and σ^{66} was clear (t = 3.633), as LacZ levels approached those of the positive control (GCN4 leucine zipper domains; 'zip'). Interaction between σ^{66} and $RsbW_{Ct}$ was observed in both cloning permutations of the BACTH assay, and expression of σ^{66} did not artificially activate the cAMP dependent reporter LacZ cassette in the absence of an $RsbW_{Ct}$ fusion protein (**S1 Fig**).

To validate the BACTH screen, molecular interactions were monitored in real time by surface plasmon resonance (SPR). Recombinant, purified σ-factors (σ^{66}, σ^{28}, and σ^{54}) were

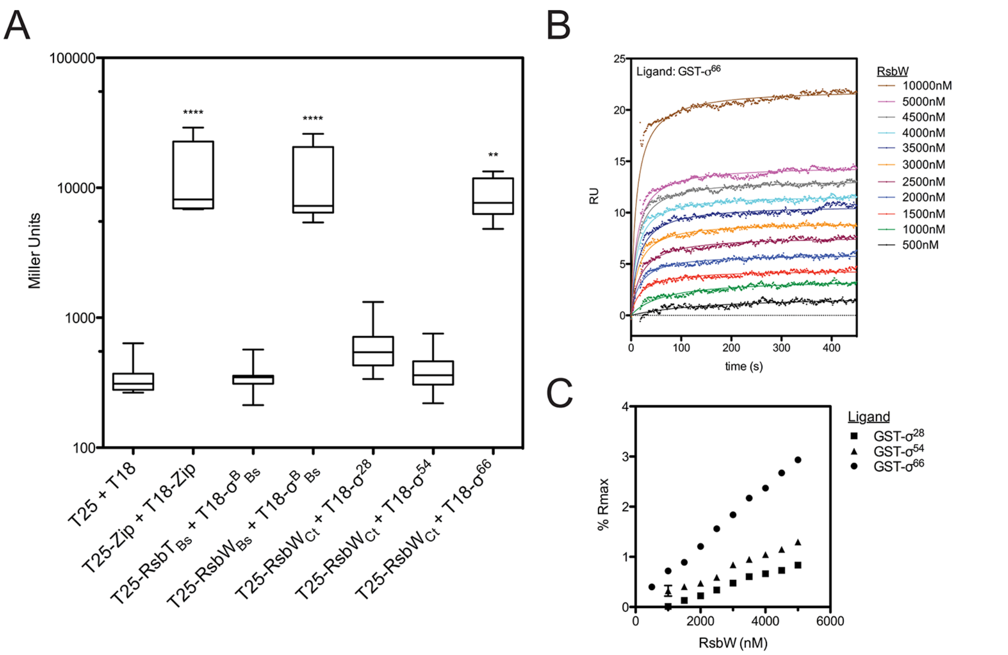

Fig 1. $RsbW_{Ct}$ associates with σ^{66}, but not σ^{54} or σ^{28}. Interactions between $RsbW_{Ct}$ and the chlamydial σ-factors were screened by BACTH (A). Mean β-galactosidase activity is displayed in Miller Units (MU). Each box represents the upper/lower quartiles transected by the arithmetic mean (N = 9). Whiskers represent the 5–95% confidence interval (** represents p<0.01, **** represents p<0.0001; One-way ANOVA with Bonferroni's post-test against the negative control). SPR analysis confirmed interaction of $RsbW_{Ct}$ and σ^{66}. Overlay of sensorgrams from a single flow-cell in which σ^{66} had been immobilized prior to injection of increasing concentrations of $RsbW_{Ct}$ show a dose-dependent response (B). Flowcells in which σ^{54} and σ^{28} were immobilized were assessed in parallel. Equilibrium rates were calculated by curve fitting and these values were transformed by the theoretical maximum binding for each ligand (Rmax). Percent of Rmax was plotted as a function of $RsbW_{Ct}$ concentration (C). Error bars (typically miniscule) represent the 95% confidence interval of the Req derived from curve fitting analysis. The overlay and Req vs. [$RsbW_{Ct}$] plots are representative of 3 experiments.

doi:10.1371/journal.ppat.1005125.g001

immobilized to consecutive flowcells of an individual CM5 sensorchip, before the chip was charged with $RsbW_{Ct}$ analyte at various concentrations. The change in response units (RU) over time, relative to a reference flowcell (immobilized Glutathione-S-transferase; GST), served as a function of binding between the ligand (sigma factor) and analyte ($RsbW_{Ct}$). Curves of best fit were applied to RU sensorgrams and the rate of equilibrium (R_{eq}) for each curve was determined (for example, a σ^{66}-binding plot overlay is shown in Fig 1B). In order to compare the relative binding of $RsbW_{Ct}$ to the three σ-factors, R_{eq} values were normalized by the theoretical maximum RU (R_{max}) for each ligand, which was based on the original immobilization levels for each sigma factor. At every concentration tested, $RsbW_{Ct}$ bound a greater percentage of immobilized σ^{66} than either of the alternative sigma factors (Fig 1C). This trend was consistent in multiple experiments (n = 3), although the overall percentage of theoretical R_{max} bound appeared to decrease with the age of the immobilized ligand on each chip (from 1 to 3 days), indicating that the activity or conformation of the immobilized ligands decreased over time. When a GST-antibody capture system was used, in which GST-tagged sigma factors were captured by immobilized anti-GST immunoglobulin immediately prior to addition of $RsbW_{Ct}$, up to 40% of the σ^{66} R_{max} was bound at a concentration of 5 μM (S2 Fig). Thus, these data indicate that $RsbW_{Ct}$ associates with the sigma factor, σ^{66}.

Interaction of RsbW with RsbV1 or RsbV2 depends on ATP and the phosphorylation state of the antagonist

$RsbW_{Ct}$ is a kinase specific for RsbV1 and RsbV2, and the phosphorylated residues of the antagonists have been mapped to Serine-56 and Serine-55, respectively [17]. In order to gain insight into the kinetics of the kinase activity for $RsbW_{Ct}$ against RsbV1 and RsbV2, aliquots from phosphorylation reactions were removed iteratively and resolved on a polyacrylamide gel supplemented with Phos-tag, an agent which causes an electromobility shift of phosphorylated proteins. When incubated in the presence of ATP, $RsbW_{Ct}$ induced a mobility shift of both RsbV1 and RsbV2, although there was a clear distinction in the rates at which the two antagonists were phosphorylated; after only 10 minutes, 100% of RsbV1 migration was shifted, whereas only a fraction of RsbV2 was shifted after 2 hours of incubation with $RsbW_{Ct}$ (Fig 2A and 2B).

To determine the direct effects of phosphorylation on the interaction between $RsbW_{Ct}$ and antagonist, SPR was again utilized; RsbV1 and RsbV2 antagonists were immobilized onto individual flow-cells of a sensorchip and $RsbW_{Ct}$ was then supplied in the absence or presence of ATP. Without ATP, $RsbW_{Ct}$ did not bind to RsbV1 (Fig 2C) or to RsbV2 (Fig 2D). When ATP was supplied along with $RsbW_{Ct}$, both ligands produced abnormal binding curves, in which an initial increase in response gradually returned to the baseline level. Subsequent applications of $RsbW_{Ct}$ plus ATP yielded no response with either ligand. We reasoned that upon addition of ATP, $RsbW_{Ct}$ was able to associate initially with, then phosphorylate RsbV1 and RsbV2, but that phosphorylation prevented their further interaction.

To explore this hypothesis, similar SPR experiments were carried out on derivatives of RsbV1 and RsbV2, in which the phospho-accepting residues were mutated to mimic either an immutable non-phosphorylated state (serine to alanine), or a permanent phosphorylation state (serine to aspartic acid). The addition of $RsbW_{Ct}$ without ATP exhibited no binding response to any of the four antagonist derivatives (Fig 2E and 2F). Supplementation of ATP with $RsbW_{Ct}$ yielded no binding response to the RsbV1 S56D or RsbV2 S55D derivatives, suggesting that a negative charge (from the phosphate group or, in this case, the aspartic acid residue) at position 56/55, respectively, of the antagonist abrogated association with $RsbW_{Ct}$. Supplementation of ATP with $RsbW_{Ct}$ produced a typical saturation-binding curve to the RsbV1 S56A and, to a lesser extent, RsbV2 S55A antagonist derivatives. The S56A or S55A derivative antagonists gave a similar binding response on subsequent $RsbW_{Ct}$ plus ATP injections, presumably because of their inability to accept phosphorylation and, rendering them inactive. Together these data indicate that the binding of $RsbW_{Ct}$ to its RsbV1 and RsbV2 antagonists depends on both the phosphorylation state of RsbV1 or RsbV2 and ATP.

Phosphorylated RsbV1 is a substrate of the C-terminal PP2C-like domain of $RsbU_{Ct}$

Three proteins contain recognized PP2C-like phosphatase (IPR001932) domains in *C. trachomatis*: CT259, $RsbU_{Ct}$ and CT589 (Table 1). $RsbU_{Ct}$ and CT589 are paralogues, both predicted to transverse the membrane with a hypothetical domain localized in the periplasm and a PP2C-like domain localized in the cytoplasm [14,17]. In contrast, CT259 appears to consist of a single cytosolic domain (Fig 3A). When aligned with the PP2C-like domains of $RsbU_{Bs}$ and SpoIIE from *B. subtilis*, the residues involved in two Mn^{2+} coordination sites, which are essential for phosphatase activity, are conserved in $RsbU_{Ct}$, but not in CT589 [17]. CT259 appears to maintain both divalent metal coordination sites, although the first site exhibits conservative aspartic acid to glutamic acid mutations (S3 Fig).

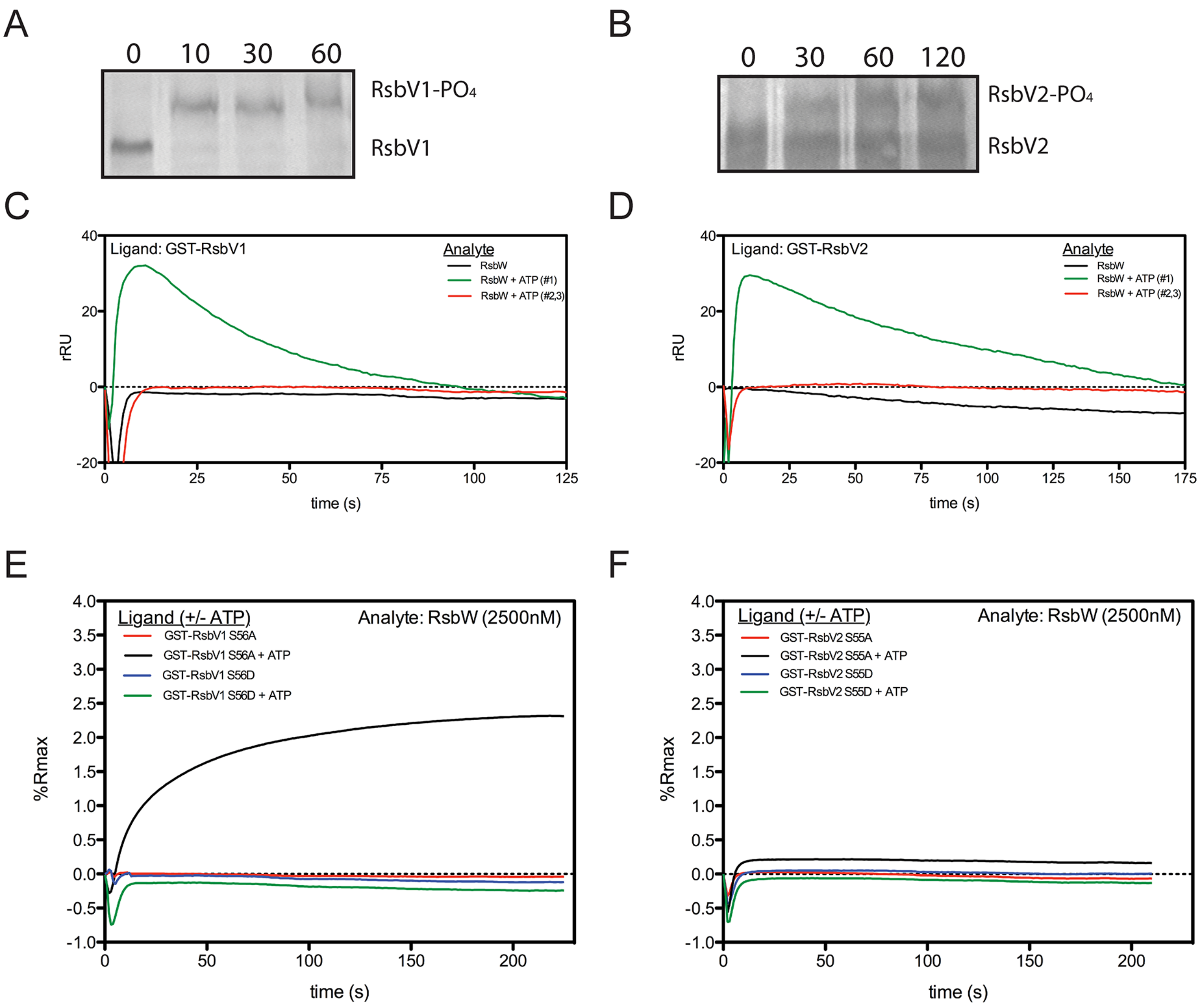

Fig 2. Association of RsbW-$_{Ct}$ with antagonist depends on ATP and the phosphorylation state of the antagonist. Purified RsbV1 (A) and RsbV2 (B) were incubated in the presence of RsbW$_{Ct}$ + ATP and monitored longitudinally (minutes) for phosphorylation, detected via electromobility shift during Phos-tag supplemented gel electrophoresis. Each panel represents two experiments in which the same results were obtained. For SPR analysis, antagonists RsbV1 (C) and RsbV2 (D) were immobilized and charged with 2500nM RsbW$_{Ct}$. in the absence (black) or presence (first attempt, green; repeated attempts, red) of ATP. Response over time was normalized by the response in an empty flow-cell. Sensorgrams shown represent two experiments (separate sensorchips) in which the same trends were observed. Derivative mutants, in which the phospho-accepting residues were changed to aspartic acid (S56D, S55D) or alanine (S56A, S55A) for RsbV1 (E) and RsbV2 (F), respectively, were tested for their ability to associate with RsbW$_{Ct}$. Serine to aspartic acid mutations, mimicking a permanently phosphorylated state, prevented the association of RsbW$_{Ct}$ in both the absence (blue) and presence (green) of ATP for both antagonists. Alanine derivatives of both antagonists, which cannot be phosphorylated and mimic a permanently active state, were bound in the presence of ATP (black), but not without ATP (red).

doi:10.1371/journal.ppat.1005125.g002

To test if the carboxy-terminal PP2C-like domain of RsbU$_{Ct}$ was an active phosphatase, the sequence corresponding to the final 258 amino acids was cloned into an expression vector for recombinant purification. This C-terminal domain of RsbU$_{Ct}$ (referred to as C-RsbU$_{Ct}$) was incubated with RsbV1 or RsbV2 that had been previously phosphorylated by RsbW$_{Ct}$. The phosphorylation states of the antagonists were distinguished by resolution on a Phos-tag supplemented acrylamide gel. Introduction of C-RsbU$_{Ct}$ caused a rapid shift from the phospho- to non-phosphorylated form of RsbV1, however phospho-RsbV2 remained phosphorylated throughout the entire time-course (Fig 3B). CT259 was also examined for phosphatase activity

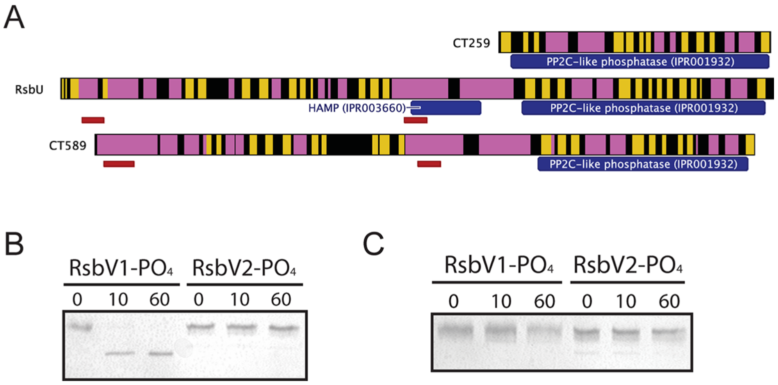

Fig 3. Proteins containing putative PP2C-like phosphatase domains in *C. trachomatis*. (A) Predicted secondary structure is shown for CT259, RsbU-$_{Ct}$ and CT589 (β-sheet represented by yellow, α-helix represented by pink). The RsbU$_{Ct}$ and CT589 paralogues are both predicted to transverse the bacterial inner membrane (represented by red bars), whereas no transmembrane domains were predicted for CT259. Phosphatase assays revealed that the RsbU$_{Ct}$ PP2C-like domain was capable of dephosphorylating RsbV1, but not RsbV2 (B). CT259 did not dephosphorylate either antagonist (C). Each panel is representative of two experiments in which the same results were obtained.

doi:10.1371/journal.ppat.1005125.g003

against the two phospho-antagonists, though no activity was observed (Fig 3C). Thus, while the C-terminal domain of RsbU$_{Ct}$ appears to maintain phosphatase activity for phospho-RsbV1, no phosphatase capable of recognizing RsbV2 was identified.

Manipulation of Rsb pathway components *in vivo*

A chlamydial shuttle vector for the controlled expression of a target cassette in *Chlamydia* was engineered in order to further investigate the role of Rsb components *in vivo*. Briefly, the shuttle vector, pGFP::SW2 [21], was modified such that the constitutive promoter driving the *gfp-cat* cassette was replaced with the tetracycline inducible promoter system from pRPF185 [22], producing pCT308-GFP (S4A Fig). The *gfp* cassette was then replaced with sequences corresponding to *rsbW* or *rsbV1* from *C. trachomatis* serovar D/UW/CX genomic DNA to make pCT1310-RsbW and pCT1310-RsbV1, respectively. These three shuttle vectors transformed *C. trachomatis* strain L2/25667R (originally lacking any plasmid; henceforth referred to as L2R) and were stable through several passages in penicillin-supplemented medium, prior to plaque purification, expansion, and density gradient harvest. Strains harboring these shuttle vectors were as resistant to penicillin as the original parent shuttle vector, pGFP::SW2 [21] with concentrations of 0.01–0.05 U/ml failing to have any effect on generation of infectious progeny, despite the same concentrations inhibiting the *C. trachomatis* L2R strain lacking any plasmid (S4B Fig). Endogenous fluorescence was not detected in *Chlamydia* harboring pCT308-GFP in the absence of ATc, but was detected upon supplementation of the growth medium (S4C Fig).

Additionally, a strain in which *rsbV1* was inactivated using insertional mutagenesis [23] was engineered. Briefly, a GII intron carrying the *aadA*-marker (to confer resistance to spectinomycin) was targeted for insertion into the 5' region of *rsbV1*, creating DFCT15 (*rsbV1*::GII[*aadA*], S5 and S6 **Figs**). The mutant strain was resistant to spectinomycin and sequencing of the disrupted *rsbV1* locus confirmed the GII intron insertion, resulting in alteration of the wild type RsbV1 sequence after 10 amino acids. Serial passage in the absence of spectinomycin followed by PCR analysis of the insertion-site using *rsbV1*-specific primers confirmed marker stability in the absence of selection, as previously observed for the GII intron carrying the *bla*-marker [23]. Consequently, experiments were performed without spectinomycin when matched with non-spectinomycin resistant strains.

RsbW$_{Ct}$ and RsbV1 affect transcription from σ^{66}-dependent promoters in *Chlamydia*

Based on the results of *in vitro* binding assays and the respective functions of homologous proteins in other bacteria, we hypothesized that RsbW$_{Ct}$ was a negative regulator of σ^{66} and that non-phosphorylated RsbV1 would act as a positive regulator of σ^{66} by antagonizing RsbW$_{Ct}$. To test this hypothesis *in vivo*, we monitored the expression of *bona fide* σ^{66}-dependent genes in our L2R transformant and *rsbV1* knockout strains. Normalization of transcript expression in *Chlamydia* is not trivial (*e.g.* [8,24]), especially considering our hypothesis of differential 'housekeeping' transcription. We tested a number of different normalization techniques that did not assume that any chlamydial gene would correlate unequivocally to the number of chlamydiae present within the sample. While use of *C. trachomatis*-specific gDNA from parallel samples as an exogenous control has been employed frequently (*e.g.* [8,25]), this type of normalization fails to account for variation in RNA loading / efficiency of reverse transcription as a source of error. We found that use of the geometric mean of *Chlamydia*-specific gDNA from parallel samples and endogenous host cell *gapdh* as a normalization factor for each sample produced a data set with the lowest cumulative intrasample error (**S7 Fig**). Primary Cq values, along with normalization calculations, are available in **S1 Dataset**. As expected, *rsbW* transcript expression was elevated in the L2R pCT1310-RsbW strain (**Fig 4A**), whereas *rsbV1* transcript expression was elevated in the L2R pCT1310-RsbV1 strain (**Fig 4B**). Transcript of *rsbV1* was not detected by RT-qPCR in DFCT15 (*rsbV1*::GII), confirming genetic analysis (**S6 Fig**). Expression of the *gfp*-cassette in L2R pCT308-GFP strain reached similar levels of the other expression cassettes (data available in **S1 Dataset**).

To test whether 'housekeeping' transcription was affected in the transformant / knockout mutant strains, we assessed the transcription of two genes with *bona fide* σ^{66}-dependent promoter systems, *16S rRNA* [26,27] and *ompA* [28,29], limiting our analysis to time-points prior to the typical RB to EB re-differentiation in order to avoid re-differentiation as a confounding variable. As predicted, σ^{66}-dependent transcription was elevated in the L2R pCT1310-RsbV1 strain, with the pooled relative expression of *ompA* and *16S rRNA* reaching a geometric mean of 2.32-fold over the control (95% CI = 1.72- to 3.11-fold). Conversely, the relative expression of σ^{66}-dependent transcription was reduced to 0.362 of control (95% CI = 0.224 to 0.583) in the *rsbV1*::GII strain. Notably, σ^{66}-dependent transcription in the L2R pCT1310-RsbW strain was reduced from the control expression (95% CI = 0.585 to 0.988), however did not reach similar repression levels as the *rsbV1*::GII strain. This negative effect has been observed in each of two previous analysis of expression experiments (summary figures shown **S8** and **S9 Figs**). The observation that the *rsbV1*::GII strain exhibits lower amounts of σ^{66}-mediated transcription compared to the RsbW$_{Ct}$ expression strain may indicate that the levels of *rsbW* achieved in these experiments were insufficient to completely overcome antagonism from endogenous RsbV1 levels, resulting in a subtle but consistent phenotype.

As a control, we also assessed whether σ^{28}-dependent transcription of *hctB* [30], was affected in the transformant / knock-out mutant strains, with the caveat that all known σ^{28}-dependent genes are transcribed during the late stages of the developmental cycle due to repression by an early-stage transcriptional repressor (EUO) [31,32]. Indeed, we were not able to detect *hctB* transcription in all samples at 12 hpi, and levels at 18 hpi neared the limit of detection. Thus, we analyzed expression of *hctB* during the late stages of the developmental cycle. At these time-points, no differential expression was observed. Taken together with the *in vitro* binding data, our results suggest RsbW$_{Ct}$ is an anti-sigma factor of σ^{66}, and that RsbV1 is a *bona fide* antagonist of the RsbW$_{Ct}$ to σ^{66} association.

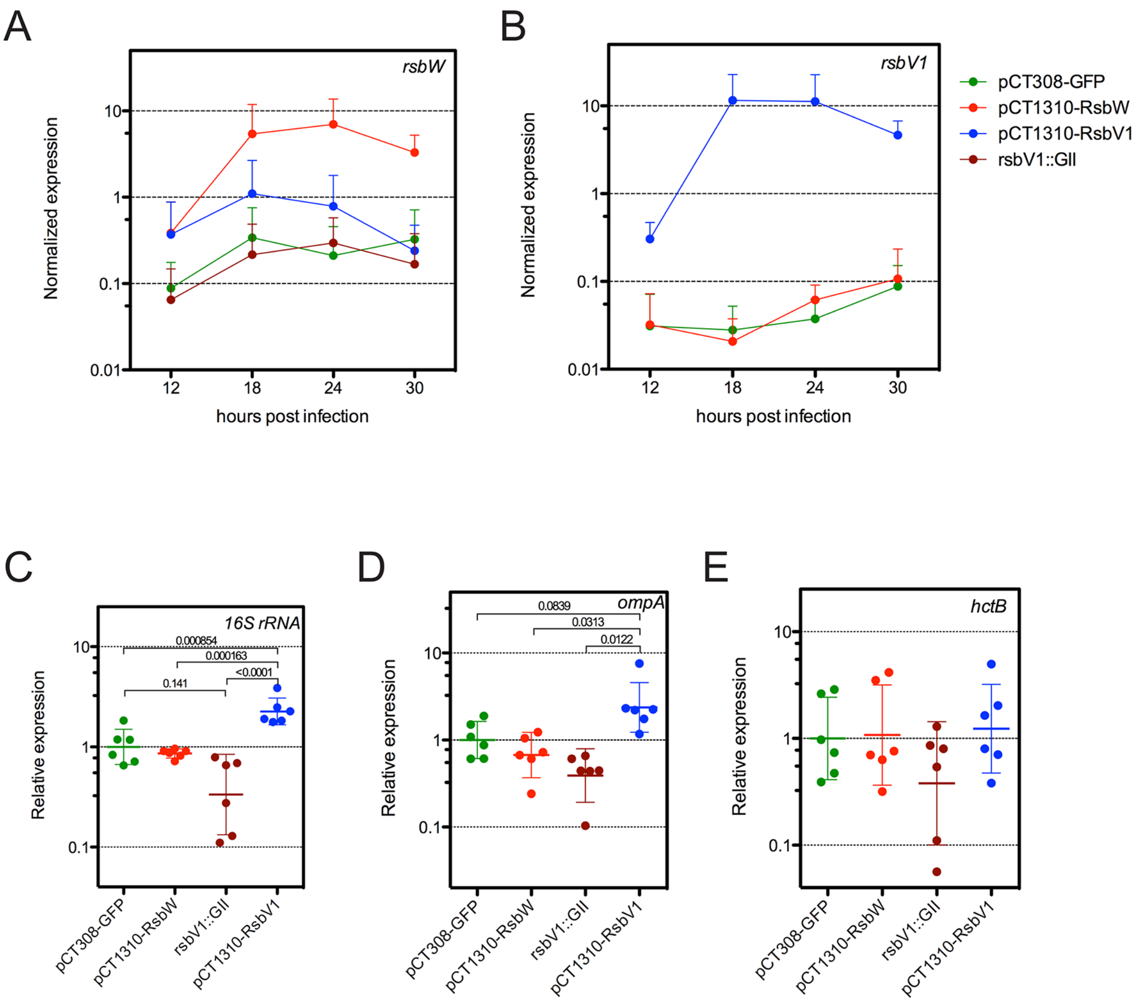

Fig 4. Analysis of σ^{66}-dependent gene expression in response to modified *rsb* component expression. Transcripts levels of *rsbW* (A) or *rsbV1* (B) were monitored in L2R strains harboring shuttle vector plasmids pCT308-GFP, pCT1310-RsbW, or pCT1310-RsbV1, as well as in strain DFCT15, in which *rsbV1* had been inactivated via transposon insertion (*rsbV1*::GII). The expression levels of target cassettes were elevated in their respective expression strains, whereas no *rsbV1* transcription was detected in the *rsbV1*::GII strain. Expression of target cassettes are shown as the mean normalized expression as a function of time (hours post infection). Error bars represent the 95% CI. Normalized expression of *16S rRNA* (C), *ompA* (D), and *hctB* (E) were calibrated to the geometric mean of the GFP-expression control. Lines indicate the geometric mean of relative expression and error bars represent the 95% CI. P-values are derived from One-way ANOVA with Tukey's multiple comparisons post-test performed in R. Transcription of *ompA* and *16S rRNA* are σ^{66}-dependent [26–29], whereas transcription of *hctB* is σ^{28}-dependent [30]. Only σ^{66}-dependent genes experienced differential expression.

doi:10.1371/journal.ppat.1005125.g004

Ectopic expression of $RsbW_{Ct}$ and RsbV1 affects chlamydial replication and development

We hypothesized that modulating the activity of the 'housekeeping' sigma factor would have detectable effects on *Chlamydia*, yet no obvious effect on growth or development was observed during the passage and selection of pCT1310-series transformants. Immunofluorescent analysis (IFA) revealed that all transformant strains exhibited a morphology consistent with acute development, and that no aberrant, enlarged chlamydial particles consistent with the persistent phenotype were observed. However, we did observe that inclusions from the L2R pCT1310-RsbV1 strain appeared much larger than the GFP or $RsbW_{Ct}$ expression strains ($p<0.0001$; One-way ANOVA, Tukey's multiple comparison post-test versus both other samples; **S10 Fig**). We reasoned that increased inclusion size observed in the L2R pCT1310-RsbV1

strain might be attributed to increased metabolic capacity upon elevated 'housekeeping' transcription, and if so, this may correlate to modulations in growth and development. To test this hypothesis, we analyzed the transformant and *rsbV1* mutant strains for genomic replication (1-step growth), recoverable infectious progeny (2-step growth), and plaque expansion.

For both 1-step and 2-step growth analysis, data was normalized by the empirical IFU input for each experiment, such that the data presented accounts for the actual number of infection events for each sample. Differential genomic replication was not overtly evident between the strains, though analysis of the area underneath each growth curve did reveal a trend that mirrored levels of σ^{66}-dependent transcription, with L2R pCT1310-RsbV1 exhibiting the highest chlamydial load, followed by L2R pCT308-GFP, and then L2R pCT1310-RsbW and the *rsbV1* mutant (Fig 5A). As a second indicator of development, recoverable infectious progeny (2-step growth) was also monitored (Fig 5B). At 24 hpi, the L2R pCT1310-RsbV1 strain exhibited a 2.3-fold increase in infectious progeny compared to control ($p = 0.0026$; One-way ANOVA with Tukey's multiple comparisons post-test), whereas L2R pCT1310-RsbW exhibited a 3.1-fold reduction ($p = 0.181$). The *rsbV1* mutant also displayed a reduction in infectious progeny that exceeded that of L2 pCT1310-RsbW ($p = 0.108$ versus control). Thus recoverable infectious progeny and, to a lesser extent, genomic replication exhibited a pattern that correlated with observed σ^{66}-dependent transcription.

However, because neither one-step or two-step growth analysis alone is a perfect measure of *Chlamydia* fitness, we chose to analyze transformant and mutant strains in a plaque expansion assay. Because plaque expansion depends on all aspects of chlamydial development (*e.g.* replication, re-differentiation, cell exit, and secondary host cell entry), the relative size of plaques can be used as an indicator of overall chlamydial fitness.

Specific locations within HeLa monolayers were inoculated with the modified *Chlamydia* strains and plaques were measured after 8–9 days, using a semi-automated procedure in FIJI [33]. An example of this process is shown in Fig 5C, in which plaques from day 9 post-inoculation are shown. In concordance with the 1-step and 2-step growth profiles, the L2R pCT1310-RsbV1 strain exhibited the highest rate of plaque expansion (Fig 5D). Moreover, the L2R pCT1310-RsbW and DFCT15 (*rsbV1*::GII) strains yielded smaller plaques than the GFP expression control. Thus, in concert with *in vitro* and transcript expression data, these results support a model in which the availability of σ^{66} is affected by relative levels of RsbW and RsbV1 and that their experimental manipulation was capable of influencing the rate at which *C. trachomatis* infection progresses in a cell culture model.

Discussion

The evidence presented in this report indicates that the primary target of the Rsb phosphoregulatory network in *Chlamydia trachomatis* is σ^{66}, the main 'housekeeping' sigma factor of the pathogen. Interaction of $RsbW_{Ct}$ with σ^{66} was shown indirectly by bacterial two-hybrid assay and directly by SPR analysis. Moreover, previous results that $RsbW_{Ct}$ is a kinase for both RsbV1 and RsbV2 *in vitro* [17] were confirmed, although there is a difference in efficiency between these phosphorylation events, with RsbV1 being the preferred substrate. $RsbW_{Ct}$ does not associate with RsbV1 or RsbV2 in their phosphorylated forms, or, interestingly, in the absence of ATP. We further observed *in vitro* phosphatase activity from the $RsbU_{Ct}$ PP2C-like domain that is specific for phospho-RsbV1, but not for phospho-RsbV2. Thus, a complete signaling module (consisting of a system phosphatase, antagonist, switch-protein, and target) has been characterized in this report. To verify the model generated from *in vitro* assays, mutant and transformant strains of *Chlamydia* were engineered for *in vivo* analysis. Elevated expression of RsbV1 correlated with the enhanced expression of *bona fide* σ^{66}-dependent transcripts

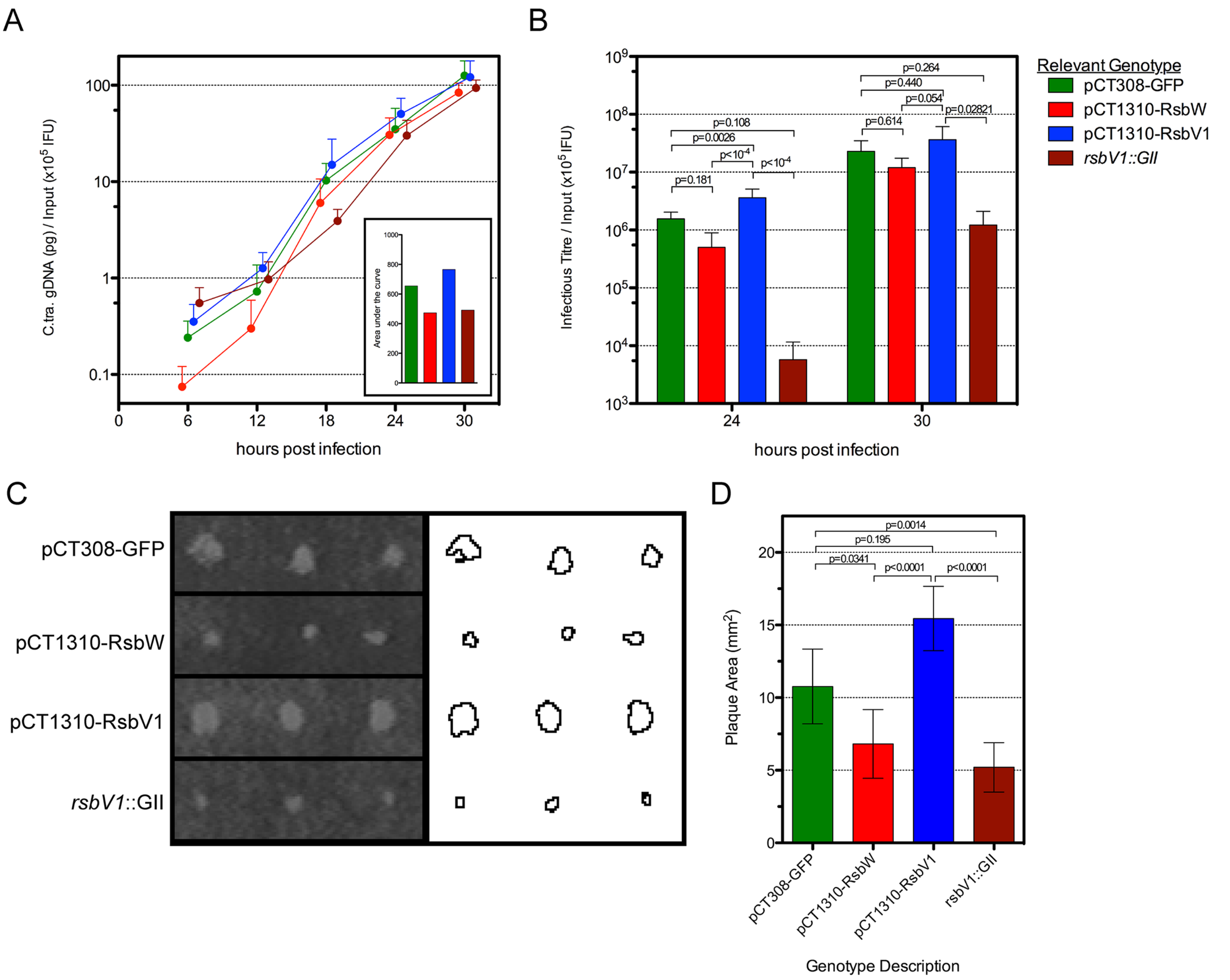

Fig 5. The effect of *rsbW* and *rsbV1* expression on genomic replication and development of infectious progeny. 1-step and 2-step growth from time-course experiments were monitored for strain L2R harboring shuttle vector plasmids pCT308-GFP (green), pCT1310-RsbW (red), pCT1310-RsbV1 (blue), or from strain DFCT15 (*rsbV1*::GII; maroon). The mean of *C. trachomatis* gDNA, normalized by the empirical IFU input (x10^5) is plotted by hours post-infection (A). The inset shows the area under each curve. The mean of recoverable infectious progeny from the same experiments was also normalized by empirical input (B). Error bars for both panels represent the 95% confidence interval. P-values derived from One-way ANOVA with Tukey's multiple comparisons test performed in R. Plaque expansion from infection foci were measured using FIJI freeware (C). Bars represent mean plaque size (mm^2) from all foci at days 8 and 9 post-infection (D). Error bars represent 95% confidence intervals. P-values derived from One-way ANOVA with Dunn's test for multiple comparisons using rank sums in R.

doi:10.1371/journal.ppat.1005125.g005

and a more rapid growth profile in multiple assays. In contrast, elevated expression of $RsbW_{Ct}$ and the inactivation of its antagonist both resulted in reduced transcription of representative σ^{66}-dependent genes and a depressed growth profile.

Taken together, these results provide a mechanism by which the Rsb network could control σ^{66} availability, and perhaps growth rate, in response to various stimuli. We postulate the following working model (Fig 6). Under steady-state conditions, the equilibrium of the Rsb network provides a molecular 'speed-limit' on σ^{66} activity and subsequently on metabolic activity

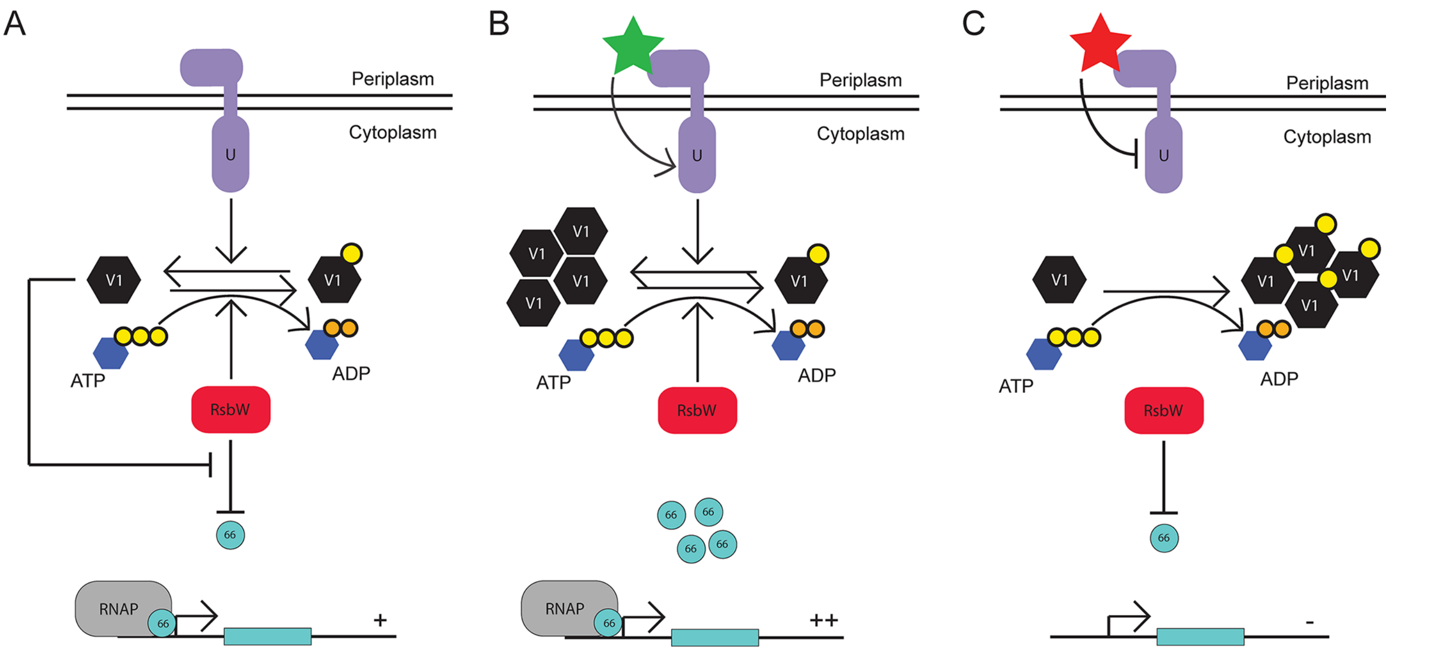

Fig 6. Proposed model for the function of the Rsb regulatory system in *Chlamydia*. Under steady-state conditions, the expression levels and activies of $RsbU_{Ct}$ (purple), RsbV1 (black), and $RsbW_{Ct}$ (red) provide an equilibrium in which σ^{66} availability is ample for normal growth and development (A). Upon an accumulation of active (*i.e*. non phosphorylated) RsbV1 via increased expression or increased activity/expression of $RsbU_{Ct}$, the equilibrium of $RsbW_{Ct}$ function shifts away from the anti-sigma factor role, allowing increased transcription of σ^{66}-promoted genes (B). Upon the decreased availability of active RsbV1 (either through reduced expression or reduced $RsbU_{Ct}$ expression/activity), the equilibrium of $RsbW_{Ct}$ function is driven towards the anti-sigma factor function, limiting the amount of σ^{66} that is available for association with the core RNAP complex (C).

doi:10.1371/journal.ppat.1005125.g006

(Fig 6A). Upon experiencing increased levels of active (*i.e*. non phosphorylated) RsbV1, the equilibrium of $RsbW_{Ct}$ function would be driven away from sequestration of σ^{66}, resulting in amplified levels of 'housekeeping' transcription. Possible inputs for this pathway would be the increased activity/expression of $RsbU_{Ct,}$ or increased expression of RsbV1 (Fig 6B). Alternatively, upon decreased expression or activity of RsbV1, the equilibrium of $RsbW_{Ct}$ would be driven towards sequestration of σ^{66}, limiting 'housekeeping' transcription and possibly restricting metabolism. Potential inputs for this shift would include decreased expression or activity of $RsbU_{Ct}$, decreased expression of RsbV1 (Fig 6C), or, intriguingly, decreased levels of ATP. A mechanism that links energy stress to decreased general levels of transcription seems plausible, however a procedure for the manipulation or even measurement of *Chlamydia* ATP levels (*i.e*. to distinguish host versus pathogen ATP pools) has not been elucidated, making exploration of this hypothesis difficult.

We were initially surprised to observe that the association between $RsbW_{Ct}$ and RsbV1 is dependent on ATP, which represents a mechanism distinct from Rsb components in the in *B. subtilis* module [34]. However, this distinction may reflect the differences in regulated targets with σ^B as an alternative sigma factor responsible for activating transcriptional response to stress, and σ^{66} as the primary sigma factor for *Chlamydia*. Under low ATP conditions in *B. subtilis*, $RsbW_{Bs}$ is not able to inactivate $RsbV_{Bs}$ such that they associate stably, liberating σ^B for the initiation of the stress response [34]. However, under low ATP conditions in *Chlamydia*, the theoretical association between anti-sigma factor and antagonist would not occur, driving the function of $RsbW_{Ct}$ towards sequestration of σ^{66} and the reduction in 'housekeeping' transcription. We postulate that the ATP-dependence of anti-sigma to antagonist interaction

theoretically must switch the Rsb system from one of stochastic activation in *B. subtilis* [35] to one of stochastic inactivation in *Chlamydia*.

This study extends the work of Hua *et al* [17], which characterized several interactions within this module, including kinase activity of $RsbW_{Ct}$ and detailed *in silico* analysis of the module members. Yet, they failed to observe any interaction between $RsbW_{Ct}$ and any of the chlamydial σ-factors in a yeast-two hybrid system. Furthermore, *in vitro* transcription assays showed no interaction between $RsbW_{Ct}$ and σ^{28}, and a separate study failed to observe any effect of $RsbW_{Ct}$ in a heterologous σ^{28}-mediated transcription assay in *Salmonella enterica* [16,17]. While we concur that σ^{28} does not interact with $RsbW_{Ct}$, we provide evidence that $RsbW_{Ct}$ indeed interacts with σ^{66}. One possible explanation for this discrepancy could be that the σ^{66}-RsbW complex was not targeted properly to the yeast nucleus due to its size, stoichiometric ratio, etc. By using a *bacterial* two hybrid reliant on generation of a cytosolic metabolite, neither complex size nor nuclear import was an issue.

Interestingly, other proteins have been described as modulators of σ^{66} function in *Chlamydia*. For instance, CT663 has been likened to the Rsd protein in *E. coli* [36], which inhibits σ^{70}-mediated transcription during stationary phase growth [37]. Another protein, GrgA, reported as a non-specific DNA binding protein, also associates with the non-conserved region of σ^{66}. By interacting with both simultaneously, GrgA enhances the transcription of σ^{66}-dependent promoter systems *in vitro* [38]. Thus, there is a clear precedent for the alteration of σ^{66} activity as an evolved strategy in *Chlamydia*. We add $RsbW_{Ct}$ to this list of σ^{66} modulators characterized *in vitro*, and additionally provide *in vivo* evidence to support its function in this role.

We would like to note that we also attempted to transform *C. trachomatis* with shuttle vectors capable of expressing RsbV1_S56A, RsbV2, RsbV2_S55A, RsbU, CT589, and CT259, of which none transformed *Chlamydia* in at least 2 attempts (in which a positive control for transformation was employed and successful). We also attempted insertional inactivation of *rsbV2*, *rsbU*, *and ct589*, but were unsuccessful. As transformation methodologies are relatively new for *Chlamydia*, we are unsure whether these observations can be attributed to effects on chlamydial fitness (*e.g.* such manipulations are lethal or at least highly detrimental) or are simply technical failures. The use of a positive control vector for each transformation, as well as the use of multiple plasmid backbones (pCT1310- and p2TK-SW2 [39]) for attempted transformations suggest the former may be more likely. Interestingly, of the genes targeted for manipulation, those that were successfully altered exhibited mild, but reproducible, phenotypic changes. Thus, it is plausible that any robust alteration in the function of Rsb module (*e.g.* the overexpression of constitutively active RsbV1 S56A) could be highly detrimental to chlamydial growth under selective cell culture conditions. Further insights into the process of chlamydial transformation should shed light onto the nature of these negative results.

What, then, is the evolutionary function of the Rsb system in *Chlamydia*? To our knowledge, there is no phenotype (occurring during acute or persistent modes of growth) that demonstrates a global modulation in σ^{66}-mediated transcription. Perhaps this is not surprising considering that beyond basic expression and availability of sigma factors, other global transcriptional repressor proteins are likely to be influential at all stages of *Chlamydia* development. For instance, the protein EUO controls transcription of both σ^{66}- and σ^{28}-dependent late genes via interaction with operating elements found in both promoter types during the early stages of infection [31]. Thus, even if the Rsb network dictated an increased availability of σ^{66}, transcription of σ^{66}-dependent late genes would be occluded by the presence of the EUO DNA-binding protein. While it is possible the Rsb module works in concert with other global regulators in order to actualize RB to EB re-differentiation, we favor a model where its function provides a molecular 'speed-limit' for housekeeping transcription (and subsequently metabolism) as a

way to avoid overuse of potentially limited nutrients. Manipulation of expression levels of $RsbW_{Ct}$ and RsbV1 via ectopic expression or insertional inactivation resulted in differential growth and development in a nutrient-replete cell culture model. The elucidation of the activities of the system components in response to inimical growth conditions could provide more resolution into the question of the evolutionary role of this system in *Chlamydia trachomatis*.

In conclusion, this report provides multiple lines of evidence that indicate the Rsb module is a *bona fide* molecular circuit capable of influencing the availability of the main sigma factor in *Chlamydia*. The potential of this network to accelerate and restrict growth rate and development substantiates it as an important pathway for further study, and may even constitute a novel target for generation of attenuated, or even accelerated, growth mutants.

Materials and Methods

Strains and parent plasmids

Chlamydia trachomatis serovar L2/25667R, pGFP::SW2 shuttle vector, and the BACTH vectors and DHM1 *E. coli* were provided by Dr. Scot Ouellette (University of South Dakota, Vermillion). The pRFP185 plasmid was received from Dr. Robert Fagan (University of Sheffield, Sheffield). *Bacillus subtilis* subspecies *subtilis* strain 168 was a received from Professor Neil Fairweather (Imperial College, London). *Chlamydia trachomatis* serovar D/UW/Cx was provided by Dr. Rey Carabeo (University of Aberdeen, Scotland).

Cloning

All PCR reactions intended for cloning purposes were performed with Phusion High-Fidelity Polymerase (NEB). Either *C. trachomatis* genomic DNA (Serovar D/UW/CX) or *B. subtilis sbsp. subtilis* 168 genomic DNA was used for template in reactions. Primer sequences are listed in S1 Table. Insert and vector ligations were either performed using traditional restriction endonuclease digest (NEB) and ligation (T4 Ligase; NEB), or in a one-step Gibson Assembly cloning reaction (NEB). Plasmids intended for recombinant protein expression are listed in S2 Table, and plasmid shuttle vectors for the transformation of *C. trachomatis* are listed in S3 Table. Site directed mutagenesis of pGEX expression vectors for the antagonist proteins was also carried out with PCR based Gibson Assembly. All plasmid insert sequences were verified by Sanger sequencing (GATC; Germany or Macrogen, USA).

Bacterial two hybrid assay

The bacterial adenylate cyclase two-hybrid method for assessment of protein-protein interaction was completed as per the instructions of the manufacturer (EuroMedex). Detailed methods are described in S1 Text. The Miller Assay determined expression of the reporter LacZ [40]. Sample groups in all graphs represent an equal number of biological replicates, indicated in figure legends.

Surface plasmon resonance

The Biacore 3000 instrument (GE Healthcare) revealed biomolecular interactions via surface plasmon resonance (SPR). Specifics for each run are described in supplemental information. CM5 sensorchips were used for all experiments. The optimal pH for pre-concentration of ligands was determined using the pH scouting wizard (Biacore 3000 software), and ligands were using the amine coupling kit. All experiments were carried out in a buffer of 10mM HEPES, 150mM NaCl, and 1mM $MgCl_2$ at 30°C. All consumables were purchased from GE Healthcare.

Protein purification

Proteins were expressed from pGEX-derived vectors in PC2 *E. coli* [41]. Soluble proteins were purified by standard techniques. Insoluble proteins were liberated from inclusion bodies using sarkosyl as described in the S1 Text. All proteins were assessed for purity via SDS-PAGE and Coomassie-Blue stain. Detailed protocols are available in S1 Text.

Bioinformatics

All DNA and protein sequence diagrams were generated using Geneious version 7.0.2, created by Biomatters. Secondary structures were predicted by the Phyre2 algorithm [42]. Transmembrane regions were predicted using the Hidden Markov Model in Geneious. InterPro domains were identified using the European Bioinformatics Database (EBI). Accession numbers listed are from the UniProtKB database.

Kinase and phosphatase assays

In vitro kinase/phosphatase assays were performed using recombinant, purified preparations of the system components and are described in detail in the S1 Text. Phos-tag reagent (Alpha Laboratories) was utilized to shift the migration of phosphorylated RsbV1 or RsbV2 during SDS-PAGE.

Chlamydia trachomatis transformation and insertional mutategenesis

Transformation of *C. trachomatis* L2/25667R (plasmid-deficient) with ectopic expression vectors was carried out, as described [21,43] with slight modifications highlighted in the S1 Text. Every transformation attempt utilized pGFP::SW2 as a positive control (100% success rate). DFCT15 was generated from the transformation of *C. trachomatis* strain L2/434/Bu with plasmid pDFTT6*aadA* as described in [23] with the exception that spectinomycin was used for selection at 500 ug/ml instead of ampicillin for mutant selection and plaque isolation of clones. The intron was targeted to insert between base pairs 28 and 29 of *rsbV1* (TCCCTTGTAAAT GAAGGATGCCTGTTTGGC—intron–CTTGTTCTTCTTTCT) in an antisense orientation. The predicted insertion efficiency values were an E-value of 0.75 and a score of 8.51. Intron retargeting was performed as directed by the TargeTron manual (Sigma-Aldrich). Both transformant and knock-out strains were considered plasmid-competent.

Chlamydia infections

HeLa cells (ATCC) at 80–95% confluence in a 6- or 12-well cluster plate were inoculated with *C. trachomatis* diluted in Hank's Balanced Salt Solution prior to centrifugation (500x*g* for 15 minutes at 20°C) and incubation at 37°C for 30 minutes. The inoculum was then aspirated before addition DMEM supplemented with 10% fetal bovine serum (FBS) and other supplements as noted. For cassette induction, anhydrous tetracycline (Sigma) was supplemented to a final concentration of 5 ng/ml at 6 hours post infection.

1-step growth analysis

C. trachomatis strains were grown on the same cluster plate for 6–30 hpi. At designated time points, infected monolayers were washed, trypsin-treated into suspension, pelleted and stored in PBS at -80°C. Total genomic DNA was extracted and diluted to a final concentration of 1 ng/ml. *Chlamydia*-specific gDNA was assayed twice using qPCR (see Quantitative PCR below) with primers amplifying a region of the hypothetical gene, *ct652.1*, or the 16S ribosomal subunit gene. Starting quantities were normalized against the empirical IFU input for each

strain in order to account for any differences the actual versus intended MOI within each sample.

2-step growth analysis

Samples intended for quantification of recoverable infectious progeny were dislodged into 1ml SPG buffer (220mM Sucrose, 10mM Na_2HPO_4, 4mM KH_2PO_4, 5mM Glutamic Acid) using sterile glass beads, and stored at -80°C. Sample infectious titers were quantified as described previously [25]. A similar process was used to determine the empirical infectivity of primary sample infections. Titers were normalized by this empirical infection rate for each strain within the individual experiment to exclude any effects of variation in stock aliquots.

Plaque expansion assay. Uninfected HeLa cells (~85–90% confluence) in 6-well cluster plates were covered with 1X DMEM containing 0.8% agarose, which was kept at 42°C until use). Agarose medium was then allowed to congeal at room temperature for ~15 minutes. Strains were diluted to a concentration of 10^7 IFU/ml. A bevelled 20μl pipette tip was briefly incubated in the inoculum and then used to puncture the gel overlay at specific sites. After inoculation, gels were overlaid with 3ml of DMEM supplemented with 15% FBS, 1μg/ml cycloheximide, 5ng/ml ATc, and 1 μg/ml gentamicin. All inoculation sites were checked for infection on day 1. Liquid overlay media were changed every 3 days. For plaque area measurement, overlay medium was carefully aspirated and the monolayers fixed by adding 10% formaldehyde in PBS to each well. After ~2 hours, gels were carefully removed and the fixed monolayers washed with PBS twice. Each well then received 0.5 ml of a 1% crystal violet in 20% ethanol solution. Monolayers were stained for ~30 minutes, prior to gentle washing of the plate in a large reservoir of ddH_2O. Images of plaques were captured from beneath the well using a Gel-Doc-It Imaging system (Bioimaging Systems) using the exact same zoom, aparature, and focus settings for each plate. Plaques were identified using FIJI [33], by adjusting the "Threshold" and then "Analyze Particles". For infection foci where no plaque was observed, a measurement of 1 pixel was recorded. Image in Fig 5C are from samples harvested on day 9 post-infection. Mean values in Fig 5D represent measurements from day 8 and 9 post-infection.

Immunofluorescent analysis

Fluorescent imaging for empirical infectivity and for recoverable infectious progeny titer was carried out on a Nikon Eclipse TE2000 epifluoresence microscope. For morphology and inclusion size analysis, images were captured using a Leica SP5 Resonant inverted confocal microscope, using identical settings for each sample. Montage images and scale bars were generated in FIJI. Inclusion sizes were determined in FIJI, using elipses to circumscribe each inclusion within a field of view from which the measurement tool provided the inclusion area (pixels).

Nucleic acid extractions

Genomic DNA extractions were prepared using the DNeasy Blood and Tissue Kit (QIAGEN). For RNA extraction, RNALater fixative was removed from infected monolayers and RNA was extracted by Trizol reagent (Life Technologies). RNA samples were treated with DNase (Turbo DNA-free kit, Applied Biosystems) for 1 hour, before DNase inactivation via the instructions of the manufacturer. cDNA was then generated from 2.5 μg of RNA sample using the Superscript III Reverse Transcriptase kit (Invitrogen), and diluted 1:8 in nuclease-free water before storage at -20°C. No Reverse Transcriptase controls were generated for all samples.

Quantitative PCR

Quantitative PCR was conducted in triplicate on the BioRad CFX96 Real-time system. Each reaction consisted of 1X Power SYBR green Mastermix, 0.45μM of each primer, and 2μl of sample in a volume of 25μl. Each run for a *Chlamydia* gene contained a standard curve of L2/25667R gDNA to assess amplification efficiency. NRT controls were performed for all cDNA samples. Equal amounts of total nucleic acid were loaded for each assay type (2 ng for gDNA, and the cDNA generated from 37.5 ng of RNA).

Transcript expression analysis

To control for both the number of *C. trachomatis* particles and for differences in reverse transcription efficiency, target gene expression was normalized by the geometric mean of an exogenous (*C. trachomatis* specific gDNA) and endogenous (host *gapdh* transcript) controls. Normalized expression was calculated as in [44], using the formula: $E_{Target}^{(-Cq[Target])}$ / Geometric mean($2^{(-Cq[GAPDH])}$, $E_{gDNA}^{(-Cq[gDNA])}$), where E represents the amplification efficiency (*e.g.* 100% = 2), Cq represents the mean cycle threshold of three technical replicates for the given run (thus, error presented is biological, not technical). All cDNA samples were assayed for contaminating gDNA with No Reverse Transcriptase (NRT) controls, of which none exhibited gDNA contamination to mathematically relevant levels.

Data analysis, statistics, and graphs

Graphs were generated using GraphPad Prism v5.0f. Statistical analysis of BACTH and SPR data were also completed with GraphPad Prism software. The data sets generated for gene expression, plaque expansion size, 1-step growth, and 2-step growth were analyzed and statistical analysis performed using R. R analysis scripts are deposited available at doi: 10.6084/m9.figshare.1466906 (1-step growth, 2-step growth, gene expression analysis) and doi: 10.6084/m9.figshare.1466907 (Plaque size analysis).

Accession numbers of relevant genes and proteins

Sigma66 (*rpoD / ct615;* P18333); Sigma28 (*fliA / ct061;* O84064); Sigma54 (*rpoN / ct609;* O84615); $RsbW_{Ct}$ (*rsbW / ct549;* O84553); RsbV1 (*rsbV_1 / ct424;* O84431); RsbV2 (*rsbV_2 / ct765;* O84770); $RsbU_{Ct}$ (*rsbU / ct588;* O84592); CT589 (*ct589;* O84593); CT259 (*ct259;* O84261); 16S rRNA (*ctr01 / 16S rRNA_1;* 884531); HctB (*hctB / ct046*; Q06280); OmpA (*ompA / ct681;* O84605); EUO (*ct446;* O84452); GrgA (*ct504*: O84512); $RsbW_{Bs}$ (*bsu04720;* P17905); $RsbV_{Bs}$ (*bsu04710;* P17903); $RsbT_{Bs}$ (*bsu04690;* P42411); SigmaB (*bsu04730*; P06574).

Supporting Information

S1 Text. Supplementary methods.
(DOCX)

S1 Dataset. Database for 1-step growth, 2-step growth, and gene expression.
(XLSX)

S1 Table. Primers used in this study.
(XLSX)

S2 Table. Expression vectors generated in this study.
(XLSX)

S3 Table. Shuttle vectors generated in this study.
(XLSX)

S1 Fig. Expression of σ^{66} does not artificially activate the cAMP-dependent promoter of *lacZ* without RsbW$_{Ct}$ complementation. The BACTH assay was performed with additional controls to ensure that activation of the cAMP dependent promoter was due to reconstitution of the AC enzyme, and not artificial activation via the heterologous expression of σ^{66} in DHM1 *E. coli*. Each data point represents the measured LacZ activity of an expanded co-transformant that had been spotted for 2 days.
(TIF)

S2 Fig. SPR analysis of captured ligands. SPR analysis of captured ligands. Immunoglobulin targeting glutathione-s-transferase (GST) was immobilized to all flow cells of a CM5 sensorchip. GST-σ^{66} and GST-σ^{28} were captured in each flow cell, prior to a GST blocking step (which created a GST only reference flowcell), and analyte charge of RsbW$_{Ct}$ at a concentration of 5000nM. The GST-only flow cell was used as a reference, and the relative response was transformed by the maximum binding theoretically possible (R_{max}) based on the amount of ligand capture.
(TIF)

S3 Fig. Multiple sequence alignment of various PP2C-like domains. The PP2C-like domains for the three chlamydial proteins were extracted and aligned with the PP2C-like domains of SpoIIE and RsbU from B. subtilis (RsbU_Bs). CT589 does not conserve residues necessary for Mn^{2+}/Mg^{2+} coordination, which are essential for phosphatase activity in PP2C-like phosphatases. CT259 is conserved at positions D199 and D238 (corresponding to D582 and D634 in RsbU$_{Ct}$), and exhibits conservative mutations at positions E27 and E69 (corresponding to D440 and D461 in RsbU$_{Ct}$), allowing theoretical ability to coordinate the Mn^{2+}/Mg^{2+} ions that are required for phosphatase activity.
(TIF)

S4 Fig. Characterization of ectopic expression shuttle vectors. Characterization of expression shuttle vectors. A) Plasmid pCT308-GFP was generated by replacing the promoter driving the *gfp-cat* cassette from pGFP::SW2 with the tetracycline inducible promoter system from pRPF185. pCT1310-RsbW and pCT1310-RsbV1 were made by exchanging the *gfp-cat* cassette with the genetic sequence of corresponding genes out of *C. trachomatis* gDNA Serovar D/UW/Cx. B) Penicillin inhibitory concentration curves were generated for stable, plaque purified transformant strains. Infections were incubated in media containing the indicated final concentration of Penicillin for 42 hours, prior to cell disruption and quantification of recoverable infectious progeny. All three transformant strains were as resistant to penicillin as the positive control pGFP::SW2. C) The pCT308-GFP strain was examined for fluorescence in non-supplemented and supplemented media (0, 1, 10, or 100 ng/ml ATc). Samples were (mock-) induced at 6 hours post infection and fixed at 28 hours post infection. *Chlamydia* was immunolabeled using convalescent human sera and a DyLight 594-conjugated secondary antibody. Images were captured by confocal microscope as described in the methods section.
(TIF)

S5 Fig. Sequence map of the intron-insertion site in DFCT15 (*rsbV1*::GII[*aadA*]). The *rsbV1* and *rsbv1*::GII(*aadA*) loci were amplified via PCR and cloned into pJET for Sanger sequencing. The intron inserted in an anti-sense orientation (relative to *rsbV1*) at position 28 (A in ATG designated as position 1) resulting in alteration of the wild type ORF after ten amino acids and a stop codon after 87 amino acids. The wild type RsbV1 is 116 amino acids of

which only the first ten would be present in the "recombinant" protein, if produced. The GII intron sequence is shown in red and is truncated for brevity.
(TIF)

S6 Fig. PCR mapping of the *rsbV1*::GII(*aadA*) locus in DFCT15. PCR was used to confirm intron insertion and orientation. Loci maps are shown in A and PCR results are shown for the intron-donor vector (B), wild type strain (C), and DFCT15 (D). Expected products for each reaction are shown in (E). PCR reactions were performed with 50 ng of genomic DNA (wild type strain and DFCT15) or 1 ng of purified plasmid DNA (pDFTT6*aadA*). PCR products were run on 0.8% agarose gels, stained with ethidium bromide, and visualized using UV trans-illumination. Images were inverted to improve contrast. Molecular weight markers (in kbp) are shown to the left of each gel image.
(TIF)

S7 Fig. Analysis of various normalization strategies on the cumulative intra sample standard deviation. We analyzed the 'intrasample' standard deviation from biological replicates (*i. e.* same strain, time-point harvest, and target gene- *ompA*, *16S rRNA*, and *hctB* only) upon various normalization strategies. We reasoned that the best normalization strategy would result in the lowest mean of intrasample standard deviation. Standard deviations were converted to % of mean for ease of comparison. Boxplots of the intrasample standard deviation are shown for the primary data (N0) and after various strategies (N05, gDNA of parallel within the subset; N06, mean gDNA of all parallel subsets; N07, *gapdh*; N12, geometric mean N07 and N05; N13, geometric mean of N07 and N06). The lowest cumulative intra sample standard deviation occurred when expression data was normalized by the host housekeeping reference gene, *gapdh* (N07). However this strategy failed to account for the number of chlamydiae present within each sample. Therefore we chose to normalize by strategy N12, which is the geometric combination of host *gapdh* expression and the *C. trachomatis* gDNA measured from a parallel sample.
(TIFF)

S8 Fig. Analysis of gene expression upon ectopic expression of *rsbW*. Transcript levels of two σ^{66}-dependent genes (*16S rRNA* and *ompA*), one putative σ^{54}-dependent gene (*ct652.1*), and one σ^{28}-dependent gene (*hctB*) from strain L2/25667R pCT1310-RsbW were monitored via qPCR, normalized to exogenous gDNA controls, and then calibrated to the normalized levels of a control strain (L2/25667R pGFP::SW2). Bars represent the geometric mean of relative expression from time-points collected during 14 to 18 hours post infection (prior to typical EB redifferentiation). Error bars represent the 95% confidence intervals. Elevated *rsbW* expression was concomitant with decreased expression of σ^{66}-transcribed genes, whereas genes transcribed by the alternative σ-factors were not differentially regulated.
(TIFF)

S9 Fig. Analysis of gene expression upon ectopic expression of *rsbW* and *rsbV1*. Transcript levels (*rsbW*, **A**; *rsbV1*, **B**; *hctB*, **C**; *16S rRNA*, **D**; *ompA*, **E**) were measured at 18 hours post infection in *C. trachomatis* L2/25667R (plasmid-free) and daughter strains harboring shuttle vectors, pCT1310-RsbW or pCT1310-RsbV1. Graphs show the expression normalized by an exogenous genomic DNA control. Relative levels of σ^{66}-dependent gene transcripts, *16S rRNA* and *ompA*, were reduced upon *rsbW* expression and increased upon *rsbV1* expression (combined mean relative expression shown in **F**), whereas a σ^{28}-dependent transcript, *hctB*, was not altered by ectopic expression of either cassette. Error bars represent the 95% confidence interval. Statistical p-values are derived from One-way ANOVA with Tukey's multiple comparisons

post-test performed in R.
(TIF)

S10 Fig. Inclusion size is altered upon ectopic RsbV1 expression. *Chlamydia trachomatis* morphology was examined in L2/25667R transformed strains harboring pCT308-GFP, pCT1310-RsbW, or pCT1310-RsbV1 (A). Samples were fixed at 28 hours post infection and images were captured using Leica SP5 confocal microscope in both red (DyLight-594 immunolabeled *Chlamydia*) and green (endogenous GFP expression) channels. Inclusions representative of the mean inclusion area are shown. Scale bars represent 10 μm. Box and Whisker plots representing inclusion area from confocal micrographs are shown in (B). Each box represents the upper/lower quartiles transected by the median inclusion size. Whiskers represent the 5–95% confidence interval, and (+) represent the mean of each group. Statistical p values are derived from One-way ANOVA with Tukey's multiple comparison post-test (**** represents $p<0.0001$).
(TIF)

Acknowledgments

We thank Dr. Scot Ouellette, Dr. Goedele N. Maertens, Dr. Robert Fagan, Professor Neil Fairweather, and Dr. Rey Carabeo for materials provided, as well as Christine Thevanesan for technical assistance with the phosphatase assays. We also thank Dr. Scot Ouellette, Dr. Goedele N. Maertens, and Dr. Katherine Thompson for critical manuscript reviews.

Author Contributions

Conceived and designed the experiments: CCT DJF SW. Performed the experiments: CCT CG DJF NML SSN. Analyzed the data: CCT DJF. Contributed reagents/materials/analysis tools: CCT CG NML DJF SSN SW. Wrote the paper: CCT DJF MOM.

References

1. CDC. Chlamydia trachomatis Genital Infections—United States, 1995. Morbidity and Mortality Weekly Report. 1997; 46: 193–198. PMID: 9072679
2. Resnikoff S, Pascolini D, Etya'ale D, Kocur I, Pararajasegaram R, Pokharel GP, et al. Global data on visual impairment in the year 2002. Bull World Health Organ. 2004; 82: 844–851. PMID: 15640920
3. AbdelRahman YM, Belland RJ. The chlamydial developmental cycle. FEMS Microbiol Rev. 2005; 29: 949–959. PMID: 16043254
4. Beatty WL, Morrison RP, Byrne GI. Persistent chlamydiae: from cell culture to a paradigm for chlamydial pathogenesis. Microbiol Rev. 1994; 58: 686–699. PMID: 7854252
5. Belland RJ, Zhong G, Crane DD, Hogan D, Sturdevant D, Sharma J, et al. Genomic transcriptional profiling of the developmental cycle of Chlamydia trachomatis. Proceedings of the National Academy of Sciences. 2003; 100: 8478–8483.
6. Nicholson TL, Olinger L, Chong K, Schoolnik G, Stephens RS. Global stage-specific gene regulation during the developmental cycle of Chlamydia trachomatis. J Bacteriol. 2003; 185: 3179–3189. PMID: 12730178
7. Shaw EI, Dooley CA, Fischer ER, Scidmore MA, Fields KA, Hackstadt T. Three temporal classes of gene expression during the Chlamydia trachomatis developmental cycle. Mol Microbiol. 2000; 37: 913–925. PMID: 10972811
8. Ouellette SP, Hatch TP, AbdelRahman YM, Rose LA, Belland RJ, Byrne GI. Global transcriptional upregulation in the absence of increased translation in Chlamydia during IFNgamma-mediated host cell tryptophan starvation. Mol Microbiol. 2006; 62: 1387–1401. PMID: 17059564
9. Timms P, Good D, Wan C, Theodoropoulos C, Mukhopadhyay S, Summersgill J, et al. Differential transcriptional responses between the interferon-gamma-induction and iron-limitation models of persistence for Chlamydia pneumoniae. J Microbiol Immunol Infect. 2009; 42: 27–37. PMID: 19424556

10. Shen L, Li M, Zhang Y-X. Chlamydia trachomatis sigma28 recognizes the fliC promoter of Escherichia coli and responds to heat shock in chlamydiae. Microbiology. 2004; 150: 205–215. PMID: 14702414
11. Mathews SA, Volp KM, Peter T. Development of a quantitative gene expression assay for Chlamydia trachomatis identified temporal expression of σ factors. FEBS Lett. 1999; 458: 354–358. PMID: 10570939
12. Douglas AL, Hatch TP. Expression of the transcripts of the sigma factors and putative sigma factor regulators of Chlamydia trachomatis L2. Gene. 2000; 247: 209–214. PMID: 10773461
13. Hughes KT, Mathee K. The anti-sigma factors. Annu Rev Microbiol. 1998; 52: 231–286. PMID: 9891799
14. Stephens RS, Kalman S, Lammel C, Fan J, Marathe R, Aravind L, et al. Genome sequence of an obligate intracellular pathogen of humans: Chlamydia trachomatis. Science. 1998; 282: 754–759. PMID: 9784136
15. Humphrys MS, Todd C, Yezhou S, Shetty AC, Chibucos MC, Drabek EF, et al. Simultaneous Transcriptional Profiling of Bacteria and Their Host Cells. PLoS One. 2013; 8: e80597. doi: 10.1371/journal.pone.0080597 PMID: 24324615
16. Karlinsey JE, Hughes KT. Genetic transplantation: Salmonella enterica serovar Typhimurium as a host to study sigma factor and anti-sigma factor interactions in genetically intractable systems. J Bacteriol. 2006; 188: 103–114. PMID: 16352826
17. Hua L, Hefty PS, Lee YJ, Lee YM, Stephens RS, Price CW. Core of the partner switching signalling mechanism is conserved in the obligate intracellular pathogen Chlamydia trachomatis. Mol Microbiol. 2006; 59: 623–636. PMID: 16390455
18. Karimova G, Pidoux J, Ullmann A, Ladant D. A bacterial two-hybrid system based on a reconstituted signal transduction pathway. Proc Natl Acad Sci U S A. 1998; 95: 5752–5756. PMID: 9576956
19. Yang X, Kang CM, Brody MS, Price CW. Opposing pairs of serine protein kinases and phosphatases transmit signals of environmental stress to activate a bacterial transcription factor. Genes Dev. 1996; 10: 2265–2275. PMID: 8824586
20. Benson AK, Haldenwang WG. Bacillus subtilis sigma B is regulated by a binding protein (RsbW) that blocks its association with core RNA polymerase. Proc Natl Acad Sci U S A. 1993; 90: 2330–2334. PMID: 8460143
21. Wang Y, Kahane S, Cutcliffe LT, Skilton RJ, Lambden PR, Clarke IN. Development of a transformation system for Chlamydia trachomatis: restoration of glycogen biosynthesis by acquisition of a plasmid shuttle vector. PLoS Pathog. 2011; 7: e1002258. doi: 10.1371/journal.ppat.1002258 PMID: 21966270
22. Fagan RP, Fairweather NF. Clostridium difficile has two parallel and essential Sec secretion systems. J Biol Chem. 2011; 286: 27483–27493. doi: 10.1074/jbc.M111.263889 PMID: 21659510
23. Johnson CM, Fisher DJ. Site-specific, insertional inactivation of incA in Chlamydia trachomatis using a group II intron. PLoS One. 2013; 8: e83989. doi: 10.1371/journal.pone.0083989 PMID: 24391860
24. Mäurer AP, Mehlitz A, Mollenkopf HJ, Meyer TF. Gene expression profiles of Chlamydophila pneumoniae during the developmental cycle and iron depletion-mediated persistence. PLoS Pathog. 2007; 3: e83. PMID: 17590080
25. Thompson CC, Carabeo RA. An optimal method of iron starvation of the obligate intracellular pathogen, Chlamydia trachomatis. Front Microbiol. 2011; 2: 20. doi: 10.3389/fmicb.2011.00020 PMID: 21687412
26. Tan M, Gaal T, Gourse RL, Engel JN. Mutational analysis of the Chlamydia trachomatis rRNA P1 promoter defines four regions important for transcription in vitro. J Bacteriol. 1998; 180: 2359–2366. PMID: 9573186
27. Tan M, Engel JN. Identification of sequences necessary for transcription in vitro from the Chlamydia trachomatis rRNA P1 promoter. J Bacteriol. 1996; 178: 6975–6982. PMID: 8955322
28. Douglas AL, Saxena NK, Hatch TP. Enhancement of in vitro transcription by addition of cloned, overexpressed major sigma factor of Chlamydia psittaci 6BC. J Bacteriol. 1994; 176: 4196. PMID: 8021204
29. Stephens RS, Wagar EA, Edman U. Developmental regulation of tandem promoters for the major outer membrane protein gene of Chlamydia trachomatis. J Bacteriol. 1988; 170: 744–750. PMID: 2448291
30. Yu HHY, Kibler D, Tan M. In silico prediction and functional validation of sigma28-regulated genes in Chlamydia and Escherichia coli. J Bacteriol. 2006; 188: 8206–8212. PMID: 16997971
31. Rosario CJ, Hanson BR, Tan M. The transcriptional repressor EUO regulates both subsets of Chlamydia late genes. Mol Microbiol. 2014; 94: 888–897. doi: 10.1111/mmi.12804 PMID: 25250726
32. Rosario CJ, Tan M. The early gene product EUO is a transcriptional repressor that selectively regulates promoters of Chlamydia late genes. Mol Microbiol. 2012; 84: 1097–1107. doi: 10.1111/j.1365-2958.2012.08077.x PMID: 22624851

33. Schindelin J, Arganda-Carreras I, Frise E, Kaynig V, Longair M, Pietzsch T, et al. Fiji: an open-source platform for biological-image analysis. Nat Methods. 2012; 9: 676–682. doi: 10.1038/nmeth.2019 PMID: 22743772

34. Alper S, Dufour A, Garsin DA, Duncan L, Losick R. Role of adenosine nucleotides in the regulation of a stress-response transcription factor in Bacillus subtilis. J Mol Biol. 1996; 260: 165–177. PMID: 8764398

35. Locke JCW, Young JW, Fontes M, Hernández Jiménez MJ, Elowitz MB. Stochastic pulse regulation in bacterial stress response. Science. 2011; 334: 366–369. doi: 10.1126/science.1208144 PMID: 21979936

36. Rao X, Deighan P, Hua Z, Hu X, Wang J, Luo M, et al. A regulator from Chlamydia trachomatis modulates the activity of RNA polymerase through direct interaction with the beta subunit and the primary sigma subunit. Genes Dev. 2009; 23: 1818–1829. doi: 10.1101/gad.1784009 PMID: 19651989

37. Jishage M, Ishihama A. Transcriptional organization and in vivo role of the Escherichia coli rsd gene, encoding the regulator of RNA polymerase sigma D. J Bacteriol. 1999; 181: 3768–3776. PMID: 10368152

38. Bao X, Nickels BE, Fan H. Chlamydia trachomatis protein GrgA activates transcription by contacting the nonconserved region of σ66. Proc Natl Acad Sci U S A. 2012; 109: 16870–16875. doi: 10.1073/pnas.1207300109 PMID: 23027952

39. Agaisse H, Hervé A, Isabelle D. A C. trachomatis Cloning Vector and the Generation of C. trachomatis Strains Expressing Fluorescent Proteins under the Control of a C. trachomatis Promoter. PLoS One. 2013; 8: e57090. doi: 10.1371/journal.pone.0057090 PMID: 23441233

40. Miller. JH. A short course in bacterial genetics. Plainview, N.Y.:: Cold Spring Harbor Laboratory Press; 1992.

41. Shun M-C, Raghavendra NK, Vandegraaff N, Daigle JE, Hughes S, Kellam P, et al. LEDGF/p75 functions downstream from preintegration complex formation to effect gene-specific HIV-1 integration. Genes Dev. 2007; 21: 1767–1778. PMID: 17639082

42. Kelley LA, Sternberg MJE. Protein structure prediction on the Web: a case study using the Phyre server. Nat Protoc. 2009; 4: 363–371. doi: 10.1038/nprot.2009.2 PMID: 19247286

43. Song L, Carlson JH, Whitmire WM, Kari L, Virtaneva K, Sturdevant DE, et al. Chlamydia trachomatis plasmid-encoded Pgp4 is a transcriptional regulator of virulence-associated genes. Infect Immun. 2013; 81: 636–644. doi: 10.1128/IAI.01305-12 PMID: 23319558

44. Vandesompele J, De Preter K, Pattyn F, Poppe B, Van Roy N, De Paepe A, et al. Accurate normalization of real-time quantitative RT-PCR data by geometric averaging of multiple internal control genes. Genome Biol. 2002; 3: RESEARCH0034.
